# Exploring variation in implementation of multifactorial falls risk assessment and tailored interventions: a realist review

**DOI:** 10.1186/s12877-023-04045-3

**Published:** 2023-06-21

**Authors:** Natasha Alvarado, Lynn McVey, Judy Wright, Frances Healey, Dawn Dowding, V-Lin Cheong, Peter Gardner, Nick Hardiker, Alison Lynch, Hadar Zaman, Heather Smith, Rebecca Randell

**Affiliations:** 1 Wolfson Centre for Applied Health Research, Bradford, UK; 2 University of Leeds, Leeds, West Yorkshire UK; 3 University of Manchester, Manchester, UK; 4 Leeds Teaching Hospitals NHS Trust, Leeds, UK; 5 University of Huddersfield, Huddersfield, UK; 6 Manchester University NHS Foundation Trust, Manchester, UK; 7 University of Bradford, Bradford, UK; 8 Leeds Office of NHS West Yorkshire Integrated Care, Leeds, UK

**Keywords:** Falls, Falls prevention, Risk assessment, Realist review, Patient participation

## Abstract

**Background:**

Falls are the most common safety incident reported by acute hospitals. In England national guidance recommends delivery of a multifactorial falls risk assessment (MFRA) and interventions tailored to address individual falls risk factors. However, there is variation in how these practices are implemented. This study aimed to explore the variation by examining what supports or constrains delivery of MFRAs and tailored interventions in acute hospitals.

**Methods:**

A realist review of literature was conducted with searches completed in three stages: (1) to construct hypotheses in the form of Context, Mechanism, Outcome configurations (CMOc) about how MFRAs and interventions are delivered, (2) to scope the breadth and depth of evidence available in Embase to test the CMOcs, and (3) following prioritisation of CMOcs, to refine search strategies for use in multiple databases. Citations were managed in EndNote; titles, abstracts, and full texts were screened, with 10% independently screened by two reviewers.

**Results:**

Two CMOcs were prioritised for testing labelled: Facilitation *via* MFRA tools, and Patient Participation in interventions. Analysis indicated that MFRA tools can prompt action, but the number and type of falls risk factors included in tools differ across organisations leading to variation in practice. Furthermore, the extent to which tools work as prompts is influenced by complex ward conditions such as changes in patient condition, bed swaps, and availability of falls prevention interventions. Patient participation in falls prevention interventions is more likely where patient directed messaging takes individual circumstances into account, e.g., not wanting to disturb nurses by using the call bell. However, interactions that elicit individual circumstances can be resource intensive and patients with cognitive impairment may not be able to participate despite appropriately directed messaging.

**Conclusions:**

Organisations should consider how tools can be developed in ways that better support consistent and comprehensive identification of patients’ individual falls risk factors and the complex ward conditions that can disrupt how tools work as facilitators. Ward staff should be supported to deliver patient directed messaging that is informed by their individual circumstances to encourage participation in falls prevention interventions, where appropriate.

**Trial registration:**

PROSPERO: CRD42020184458.

**Supplementary Information:**

The online version contains supplementary material available at 10.1186/s12877-023-04045-3.

## Background

Falls are the most common safety incident reported in acute hospitals [1] and can cause both physical (e.g. hip fractures, soft tissue injuries) and non-physical harm (e.g. reduced confidence, fear of falling). Falls typically result from multiple interacting causes such as age-related physiological changes, cognitive impairment, medical causes, medications, and environmental hazards [2]. Traditionally, falls prevention strategies have used falls risk prediction tools [3]. These tools stratify patients e.g., as high, medium, or low risk of falls, with standardised interventions implemented for individuals stratified as high risk. However, falls risk prediction tools have issues such as weak predictive value [4] and poor discrimination, where almost all older patients are identified as high risk, and a score provides reassurance that action is being taken when it is not [5]. Since 2014, the National Institute for Health and Care Excellence (NICE) in England has recommended that patients in acute hospitals aged 65 and older, and patients aged 50–64 judged by a clinician to be at higher risk of falling receive a multifactorial falls risk assessment (MFRA) and tailored interventions [6]. Instead of stratifying patients according to risk, a MFRA is conducted to identify individual falls risk factors e.g., cognitive impairment, continence, problems with vision, medications that increase the risk of falls, medical causes of falls, problems with strength or balance, and whether the patient has appropriate footwear, with interventions delivered that address, improve, or manage individual risks during their hospital stay.

Evidence suggests that multifactorial approaches may help reduce incidents of falls [1, 7], but there is substantial unexplained variation between hospitals in implementation of MFRAs and associated care plans, e.g., the 2022 National Audit of Inpatient Falls (NAIF) report noted that 34% of hospitals are still using falls risk prediction tools and that, of patients who required one, a mobility care plan was in place for 90%, a continence care plan for 78%, and a delirium care plan for 61% [8]. This realist review sought to explore why there is this variation by examining what supports and constrains implementation of MFRAs and interventions tailored to individual falls risk factors.

## Methods

Realist review considers intervention impacts as highly dependent on context [9, 10], and, therefore, is useful for exploring interventions where implementation and impact vary. The aim is to construct, test, and refine programme theories configured as Context Mechanism Outcome configurations (CMOcs). Mechanisms underpin how the programme is expected to work; in this review, mechanisms were conceptualised as how and why staff and patients reason about and respond to resources offered to support delivery of MFRAs and tailored interventions; for example, training in falls prevention (resource) might be offered to staff with the intention of increasing their knowledge or confidence (responses) to deliver the recommended practices. The settings of interest were wards that cared for adult patients in acute hospitalsand Context was explored as the circumstances within this setting that influence (support or constrain) mechanisms in action. Outcomes are impacts (expected and unexpected) of interactions between Mechanisms and Contexts [11]. In this review, the outcomes of interest were the extent to which MFRA and tailored interventions were delivered as intended.

CMOcs were constructed in three stages, (1) literature searches were conducted to develop an Initial Programme Theory (IPT) and tentative CMOcs using practitioner explanations about how and why falls prevention practices are delivered, (2) the breadth and depth of literature to test the CMOcs was scoped from a search in Embase, (3) search strategies were edited and translated for searching multiple databases to test CMOcs, and prioritised by the project advisory and lay group. These groups included clinicians and academics with expertise in falls prevention and realist research methods, and lay people, most of whom had experienced a fall or were a relative of someone who had experienced a fall in hospital. Search strategies were developed in collaboration with an Information Specialist (JW) with expertise in realist reviews. The RAMESES reporting guidelines were followed, and the review protocol was published [12] and registered on PROSPERO: CRD42020184458.

### Construcitng an intiial programme theory (stage 1)

**Fig. 1:**
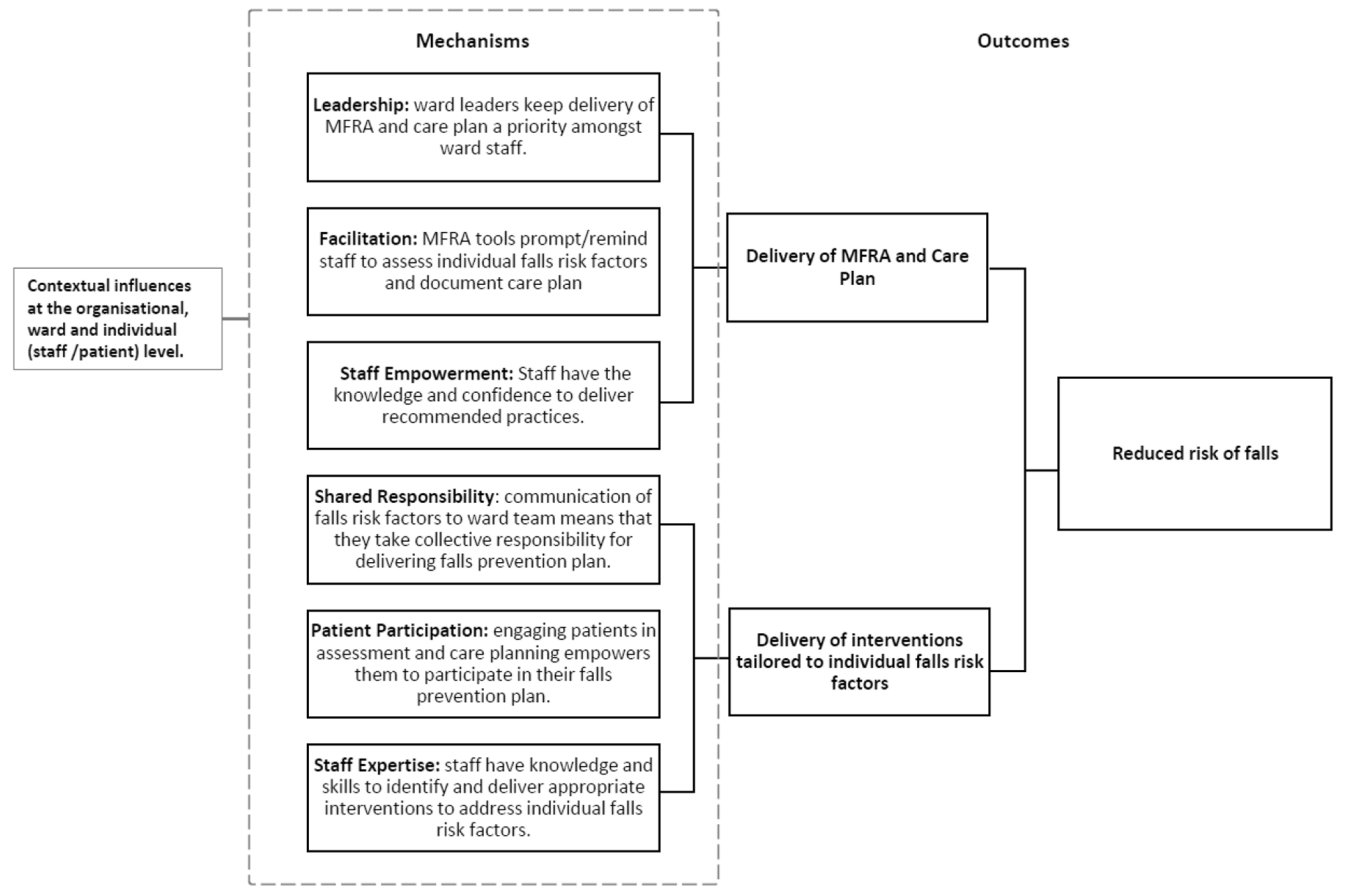
Initial programme theory depicting mechanisms that support implementation of falls prevention practices

Searches of published and grey literature, including professional and trade journals, were conducted to elicit practitioner explanations about how and why certain resources lead to delivery of falls prevention practices, and what supports or constrains this process (see Appendix i for search 1 strategies used in all sources). Six mechanisms were elicited from the literature analysis and grouped as those that explained (1) delivery of an MFRA and falls prevention care plan, and (2) delivery of interventions that addressed individual falls risk factors. The six mechanisms are presented as an Initial Programme Theory (IPT) in Fig. 1.

Six tentative CMOcs were derived from the IPT. However, literature searching for CMOc testing was an iterative process, during which an assessment was made of both the number of records returned and relevance of abstracts found per CMOc. Based on this iterative assessment and in discussion with the project advisory and lay groups, two CMOcs labelled Facilitation and Patient Participation were prioritised for testing and refinement because they were considered significant in underpinning successful delivery of falls prevention practices, testable using the literature available, and allowed explanation-building around the two outcomes of interests. The two CMOcs were expressed as follows:

#### Facilitation

In contexts where nurses are educated about falls risk factors and prevention practices (C), if MFRA tools (including Health Information Technology (HIT)) that reflect best practice recommendations are relatively quick and easy to use and are easily integrated into existing workflows, staff will complete them with patients because they facilitate delivery of recommended practice (M), helping to ensure that all patients eligible receive a comprehensive, MFRA to have their falls risk factors identified and receive appropriate interventions (O).

#### Patient participation

Where patients have the capacity to engage in the MFRA process (C), and where staff involve patients and carers in the assessment and care planning process, taking into consideration their needs and preferences, then patients will understand their strategy and have confidence to participate in specific interventions (where they are capable and able to do so) (M), thereby collaborating with ward staff to reduce their falls risk factors (O).

### CMOc testing (stages 2 and 3)

Two searches plus an update search were conducted to test CMOcs. All searches used a combination of subject headings and free text words, did not limit results to language or date of publication, and were peer-reviewed by a second information specialist. The first search was conducted in Embase to gauge the size and relevance of literature for testing all six CMOcs (see strategy 2.1 in Appendix ii). The second search was conducted in May 2021 and was run on nine databases to draw on a wider coverage of academic journals and grey literature. Table 1 lists the information resources used, and search strategies for all resources are in Appendix ii. In August 2022, searches for the Facilitation and Patient participation CMOcs were re-run on all databases except NICE Evidence which ceased in April 2022.

**Table 1:** Databases searched to test the IPT

	Information Resource
Published literature	CINAHL (EBSCOhost) Ovid MEDLINE(R) ALL 1946 to May 05, 2021 Arts & Humanities Citation Index (Web of Science) 1975+ Science Citation Index-Expanded (Web of Science) 1900+ Social Sciences Citation Index (Web of Science) 1900+ Emerging Sources Citation Index (Web of Science) 2015+
Grey literature	NICE Evidence Conference Proceedings Citation Index- Science (Web of Science) 1990 + Conference Proceedings Citation Index- Social Science & Humanities (Web of Science) 1990+

### Selection and appraisal of manuscripts

Records from searches were screened and sorted into folders for each CMOc area in EndNote. Duplicates were removed across the searches, however, records relevant for more than one CMOc were saved in all their appropriate CMOc search groups to ensure the full set of potentially relevant records were available to screen in each CMOc. Citations and abstracts were screened for inclusion by two researchers (NA and LM) using the following inclusion criteria:

- Study takes place in acute or rehabilitation hospitals.
- The intervention is about multifactorial falls risk assessment and/or falls prevention interventions. *Whilst a clear theoretical divide can be made between traditional risk stratification and MFRA tools, hybrid approaches, with the use of a risk stratification tool plus some tailoring may be seen in the literature and in practice and were included in the review.*
- The study reports empirical data.
- The study includes evidence that can contribute to testing a CMOc.

Researchers screened 10% of citations/abstracts. They then met to discuss their experiences and any discrepancies in decision making, to come to agreement over how the criteria should be applied for the remaining citations.

### Analysis and quality appraisal

Study details, including methods, settings, samples and intervention description were extracted into a Summary Table, see Appendix iii. Researchers examined outcomes relevant to each CMOc, e.g., for Facilitation, some studies measured compliance with delivery of an MFRA and care plan. To understand why there was variation in impact a thematic framework was constructed in NVivo (qualitative data analysis software) to extract data relevant to each CMOc. For example, for Facilitation, a theme heading was *Tool Type/Content* that captured differences across studies in the resources staff used to support delivery of a MFRA, with sub-themes including alerts and reminders. The included manuscripts were appraised using the Mixed Methods Appraisal Tool (MMAT) [13] and an appraisal of the quality and weight of evidence supporting CMOc refinements was made using GRADE-CERQual [14], see Appendix iv for GRADE-CERQual Statements.

## Results

**Fig. 2:**
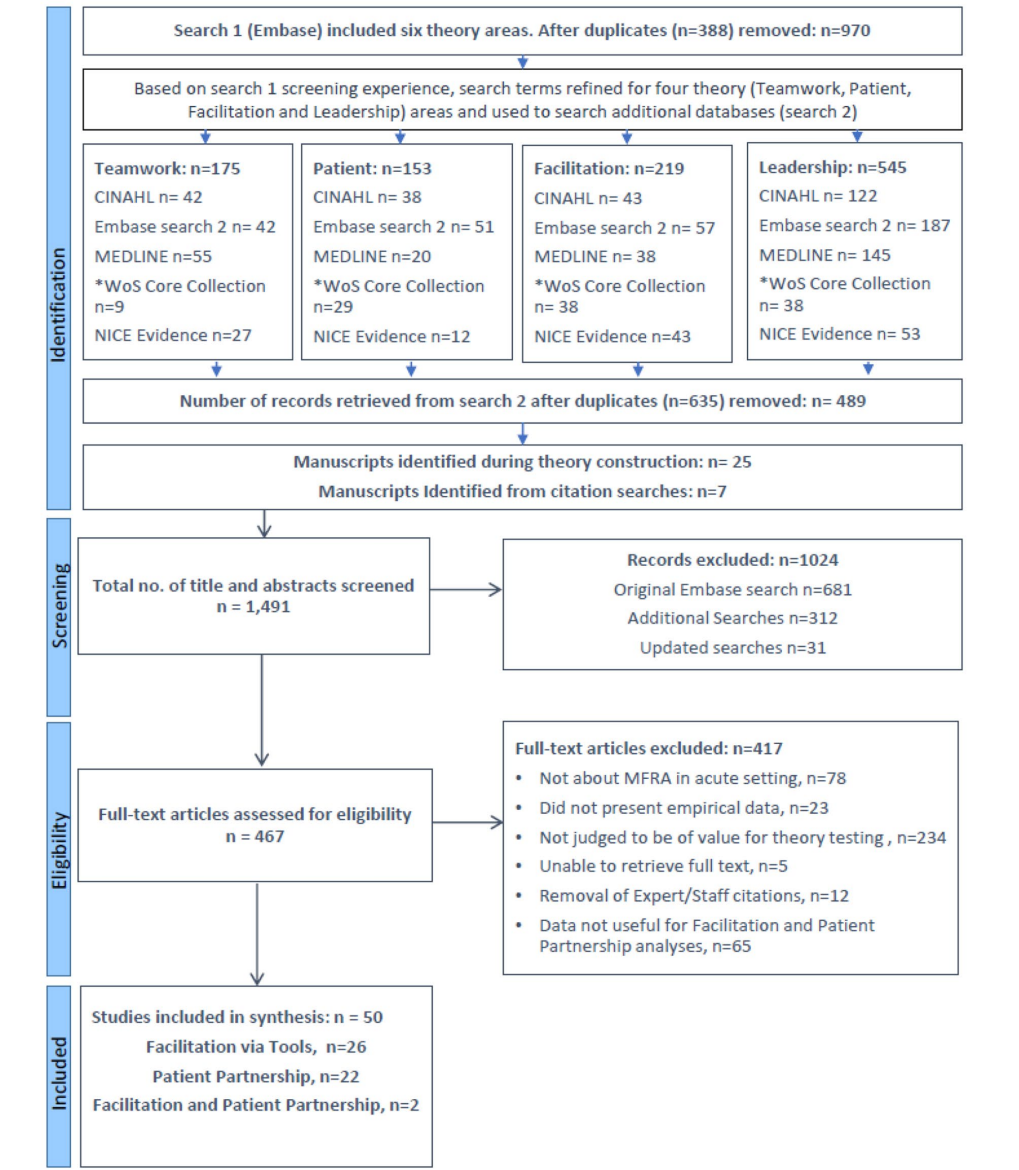
PRISMA diagram

In total 1,491 citations and abstracts and 467 full texts were screened, resulting in the inclusion of 28 manuscripts for Facilitation [15–42] and 24 manuscripts for Patient Participation [23, 25, 43–64], see Fig. 2 for PRISMA diagram. The studies were conducted in a range of countries and encompassed a range of methods, many were quality improvement projects describing examples from practice, see Appendix iii for summary of study details.

Findings are presented below by CMOc and the questions addressed in the analysis.

### CMOc 1: Facilitation ***via*** tools

#### How are MFRA tools designed to facilitate falls risk assessment?

Ten studies described use of published tools such as the Morse Fall Scale [16, 18, 23–25, 32], the Memorial Emergency Department Fall Risk Assessment Tool (MEDFRAT) [33], the Fall Risk for Older People (FROP) [37] and KINDER 1 [19, 41]. Seven studies described locally developed tools e.g., developed through review of the falls literature and/or identifying common risk factors on a particular unit [17, 21, 26, 28, 34–36]. In nine studies, it was unclear whether the assessment tool was publicly available or locally developed [15, 20, 22, 27, 29, 31, 37, 40, 42].

MFRA tools offered a structure of items to guide identification of individual falls risk factors, but the number and type varied. See Table 2 for examples of how tools compared against items recommended in the NICE guidance.

**Table 2:** Example of falls risk items included in different tools

	NICE (2013)	MORSE Fall Scale	KINDER 1	Site specific
1	Cognitive Impairment	Mental status	Altered mental state	Disorientated
2	Continence	N/A	N/A	Requires assistance toileting
3	Falls history	History of falling	Presented to ED due to fall	Two falls in the last 12 months
4	Footwear	N/A	N/A	N/A
5	Health problems	Secondary diagnosis (more than two medical diagnoses)	N/A	N/A
6	Medication	N/A	N/A	High risk medication Patient taking more than four medications
7	Postural instability, Mobility/balance	Gait	Impaired mobility	Unsteady gait
8	Syncope syndrome	N/A	N/A	N/A
9	Visual impairment	N/A	N/A	N/A
Other	N/A	Ambulatory aid IV / No IV	Age 70 or older Nurse judgement	At risk behaviours

Table 2 indicates how falls risk factor items differ depending on the tool. Cognitive impairment, mobility and history of falls are commonly included items. However, items that appear similar may not prompt the same information e.g., falls history within the NICE guidance refers to how, where, when, and why falls occur which might identify syncope or other treatable causes, whilst similar items in the tools listed require a ‘yes/no’ response.

#### How are MFRA tools designed to facilitate intervention delivery?

Some studies described how tools were designed to support decision-making in choice of intervention in response to risk factors identified e.g., providing guidance about interventions to implement in response to falls risk factors [17, 26, 28, 29, 35]; some hybrid tools also recommended standard intervention bundles for patients assessed as high risk. One study focused on intervention delivery, providing quick reference guides, organised by risk factor area, to inform choice of intervention [40]. Several studies used visual tools e.g., posters, to remind staff, patients, and carers of interventions in place for individual falls risk factors [23–25, 27, 37, 38].

#### The role of health information technology

Nine studies described that the MFRA and care plans were integrated into the Electronic Healthcare Record (EHR), digitising documentation of falls prevention practices [15, 19, 22, 32, 33, 35, 36, 41, 42]. Manuscripts that focused on the role of HIT included assessment of the impact of digitising MFRA documentation [22, 42], automating parts of the assessment and/or care planning process e.g., automatically generating a care plan with interventions linked to the falls risk factors identified during the assessment [18, 20, 23–25, 37, 38] and an evaluation of EHR alerts that notified staff to incomplete documentation [31].

#### To what extent were falls risk assessments and interventions delivered?

Twelve studies assessed delivery of a MFRA as documented in clinical records with improvement post-intervention (intervention referring to MFRA tools that were often introduced as part of a multifaceted improvement strategy, see Appendix iii for study details) reported in 11 studies [15, 18, 20–22, 28, 29, 31, 32, 35, 42], encompassing paper-based and HIT tools. One study found MFRA delivery was consistent pre- and post-intervention [19].

Documentation of a care plan in clinical records was reported in seven studies [15, 18, 31, 32, 35, 40, 42]. Lytle et al. [31] reported that documentation of risk assessments improved significantly, in response to electronic alerts, whilst care plans did not. Wu et al. [42] showed that digitisation improved documentation of practice but care plans were not documented for all patients assessed as high risk of falls.

Three studies reported use of targeted interventions, two of which demonstrated an improvement post-intervention [32, 35] and one a decline in two out of three wards studied [15]. Titler et al. [40] reported significant improvement (p < 0.001) for use of specific interventions including for mobility, toileting, and cognition, but not for medications. In Carroll et al., [18] documentation of a MFRA and care plan improved, whilst documentation of intervention delivery did not.

Three studies measured adherence displaying a bed side poster generated from Fall TIPS [23–25], a HIT intervention that aimed to involve patients, with their families and carers, in the assessment and care planning process to overcome patient non-adherence to falls prevention strategies.

In summary, whilst documentation of a MFRA improved quite consistently across studies (where reported), there was variation in impact regarding documentation of care plans and interventions delivered.

#### Why and in what circumstances do tools facilitate falls prevention practices?

There was a paucity of data detailing staff experiences using MFRA tools, although some studies provided an explanation as to why particular tools were chosen, e.g., to reduce variation in the assessment content by providing a standardised structure (32), and to improve risk identification by introducing items tailored to the patient population [17, 19, 33]. Some authors suggested tools may work simply by drawing staff attention to required practices, acting as a prompt [21, 26, 29]. To work in this way, evidence indicated that tools, paper-based and HIT-based, need to be clearly visible to staff in their work processes [15, 31, 33]. Automation of practices *via* HIT removed task loads from clinical staff – automatically linking falls risk factors to interventions and generating a care plan - but introduced novel manual work such as displaying and updating bedside posters, that brought new challenges e.g., remembering to move posters when patients swapped beds [38]. One study suggested new manual tasks may be seen as a competing priority for which staff do not have time [27].

Educational strategies, such as training and feedback, were highlighted as supports for tool use because they raised staff awareness of the tool, increased their knowledge of falls prevention practices, and evidenced the importance of following tool guidance [15, 17, 29, 30]. However, it was not possible to distinguish the impact of individual interventions as studies often incorporated multiple strategies to improve practice. Furthermore, HIT was found to introduce additional training needs e.g., in one study staff were motivated to use HIT but required more training than had been provided, to use the technology itself [38].

There was some data to suggest that staff responded well to tools that provided space to document clinical judgement, particularly where stratification (a practice no longer recommended by NICE) was used. For example, a hybrid tool recommended remote video monitoring to patients stratified as high risk of falls [19]. The authors reported that staff felt empowered by a clinical judgement item to allocate this intervention to patients most in need and according to resources available. Other studies provided further insight into to the problems of stratification, e.g., in one study staff were confused over the definition of high risk patients because they did not always judge a patient to be at risk when indicators on the tool suggested that they were [31]. One study suggested that discrepancies between tool stratification of patients as high risk and nurses’ clinical judgement may help explain why care plans were not documented consistently for patients [42].

Alongside clinical judgement, the studies pointed to a number of factors that influenced the extent to which tools acted as practice facilitators e.g., changes in patient condition and transition between wards were highlighted as circumstances that may disrupt tool use and documentation of care plans [32, 38]. Lack of communication of the falls prevention plan between different professional groups and availability of physical resources, e.g., non-skid socks, may constrain delivery of interventions suggested by tools [21, 33, 34, 40]. Furthermore, hospital IT infrastructure dictated what HIT was available to staff at the ward level e.g., whether automation was available or not [23].

#### Programme theory refinement

The Facilitation analysis was used to refine the CMOcs and overarching IPT and summary statements reflecting refinements were assessed using GRADE-CerQual (see Table 3).

**Table 3:** Facilitation: Programme Theory Refinement

Context	Mechanism		Outcome	GRADE-CerQual assessment of confidence in summary statement.
	Intervention	Staff Response		
Where staff understand (through experience, or education or feedback) how and why falls prevention practices reduce falls risk factors.	MFRA tools are located visibly and intuitively in the Electronic Health Record or ward practice and offer a structure to guide identification of fall risk factors. However, assessment tools vary in type and number of assessment items.	**Reminder**: Tool draws staff attention to the tasks required e.g., completing an assessment of individual falls risk factors and prompts action.	More consistent documentation and delivery of falls risk assessments but content of assessment may differ depending on tool used by service.	**Moderate confidence** – it is likely that the review finding is a reasonable representation of the phenomenon of interest.
Ward conditions are complex – patients’ condition may change, they may swap beds or move wards, and they may require multiple interventions.	MFRA tools are located visibly and intuitively in the Electronic Health Record or ward practice and offer a structure to guide identification of fall risk factors. However, assessment tools vary in type and number of assessment items.	**Prioritisation**: Staff attention is focused on care delivery rather than documenting care processes.	Documentation of care process may be less consistent, particularly after the initial falls risk factor assessment.	**Moderate confidence** – it is likely that the review finding is a reasonable representation of the phenomenon of interest.
Staff who are educated and experienced in identifying and managing falls risk factors.	MFRA tools are visible to staff in their work routines and provide guidance for assessing risk and linking risk factors with interventions.	**Clinical Judgement**: Tool guidance does not align with clinical judgement or resources available - staff apply care according to their own judgement.	Care may not be in line with tool recommendation, but action taken to manage risks using ward resources.	**Low to Moderate confidence** – it is possible/likely that the review finding is a reasonable representation of the phenomenon of interest.
IT systems support HIT function and staff are trained and experienced with use of HIT. Where staff understand (through experience, or education or feedback) how and why falls prevention practices reduce falls risk factors.	MFRA tools are located visibly and intuitively in the Electronic Health Record or ward practice environment. Care plans, poster and information leaflet automatically generated from software.	**Automation**: Interventions to address falls risks automatically selected and documented in care plan and patient poster. Staff display poster at patient bedside and action care plan.	Reduced variation in development and documentation of care plan that links falls risks with appropriate interventions. Task load of clinical staff reduced. Falls prevention strategy more visible in poster at patient bedside.	**High confidence**: It is highly likely that the review finding is a reasonable representation of the phenomena of interest.
		**Manual work**: Staff see manual work as competing priority with other responsibilities.	Display of poster may be disrupted by patient flow e.g., between beds and wards.	

### CMOc area 2: patient participation

#### What are the characteristics of interventions designed to encourage patient participation?

Nine studies [25, 43, 46, 49, 50, 53, 54, 57, 63] examined interventions that sought to engage patients in the assessment and/or care planning process to encourage their participation in falls prevention interventions. Radecki et al. [54] and Sitzer [57] introduced tools that enabled patients to self-assess their falls risk, recognising a discrepancy between patients’ and professionals’ perception. Martin et al. [53] evaluated the Safe Recovery Programme in which ward staff and volunteers worked with patients on one or more occasions to develop personalised goals to prevent falls. Haines et al. [49] compared two approaches, one in which patients were provided with educational materials, and a ‘complete programme’ where materials were supplemented by one or more follow-ups with a physiotherapist for goal-setting and review. Three studies examined Fall Safety Agreements [43, 50, 64] e.g., Bargmann et al. [43] introduced an agreement that patients signed to confirm that they had been educated on fall risk prevention strategies, acknowledged falling could cause serious injuries and therefore agreed to ask for help to prevent falls. Five studies examined Fall TIPS [23, 25, 46, 63], an intervention in which staff, patients and their carers worked in partnership throughout the assessment and care planning processes to prevent falls. However, how patients and carers interacted with staff during these processes was not explained.

Three studies examined interventions where patient participation was encouraged during comfort rounds, also known as intentional, purposeful, or hourly rounding [44, 48, 60]. During intentional rounding, staff asked about patients’ immediate and personal needs [44, 48, 60]. Cann and Gardner [44] described their aim as moving from a ‘patient allocation’ to a ‘practice partnership’ model of care, within which intentional rounds were intended to support patients to participate more fully in their own care. Goldsack et al. [48] examined hourly rounding with an intention of decreasing call bell usage, by engaging patients as active partners and Zadvinkis [60] conducted a survey, part of which was about intentional rounding, but no information was provided about what form rounding took.

#### To what extent do patients participate in falls prevention practices?

There was limited data evidencing the extent to which patients participated in falls prevention interventions. One study reported a significant reduction in patients’ use of call bells from 1277 uses per 100,000 patient hours to 523 uses (P = < 0.001) after comfort rounds [44]. Two studies described the types of goal that were set during patient and professional interactions [49, 53]. Common goals in both studies included working more effectively with healthcare staff, identifying environmental hazards, and using appropriate aids and equipment.

A more commonly measured impact was patient knowledge. Seven studies measured patient understanding of their falls risks and care plan with varied results [23, 25, 43, 53, 54, 61, 63] e.g., Radecki et al. [54] conducted a knowledge-in-action survey which showed statistically significant improvements between baseline and intervention groups (P = 0.0007) in patient involvement in care planning. However, there was no significant difference in other questions, including whether the prevention plan was always followed. One study (examining Fall TIPS) reported that patient activation, a term that encompassed knowledge, skill, and confidence to participate in falls prevention, improved preintervention to postintervention at three sites, with the mean score improving from 63.82 (standard deviation [SD] ± 17.35) to 80.88 (SD ± 17.48), p < 0.0001. Bargmann et al. [43] used staff incentives to increase adherence to programme implementation, which was thought to have supported an increase from 30% (5 out of 17 patients) to 95% of patients correctly stating their falls risk.

#### Why and in what circumstances do patients participate in falls prevention strategies?

The studies suggested patient attitudes, beliefs and understanding about falls risks may constrain their participation in falls prevention interventions e.g., patients may be reluctant to use the call bell for fear of disturbing busy nurses [45, 53], they may not accept they are at risk of falling [53, 55, 56, 58], and patients that have had a recent fall may be more likely to engage in falls prevention than patients who have not [51]. Studies also reported intention to act e.g., asking for help using the toilet, may not be followed through if the help requested is not forthcoming and patients feel confident to act alone [59] or are unable to wait due to urgency [58]. Additionally, patients may not be physically able to participate e.g., depending on where the call bell is placed [45].

In the Safe Recovery Programme, introduced previously, Martin et al. [53] explained that individualising messages to address patient circumstances, such as those described above, may trigger participation mechanisms such as gaining permission to ask for help, empowerment to act, and increased awareness of risk. The quality of interaction between nurse and patient was highlighted as key to successful messaging. Volunteers in Martin’s study were said to have skills such as listening, teaching, and reflecting that created engaging, personalised, safe interactional spaces. Similarly, one study suggested nurses with more experience (defined as two or more years) moved the risk assessment process from ‘task mode’ to a vehicle to enhance communication and partnership that authors linked to falls reduction [54]. Based on previous experience, effectively communicating the care plan to patients was emphasised as a key component of Fall TIPS, with studies evidencing a reduction in fall rates and improvements in patient activation [23, 46, 63].

Few studies included patients with cognitive impairment [49, 51, 53, 65]. Martin et al. [53] included patients with mild cognitive impairment and explained that posters and environmental cues e.g., call bell in place, may work as reminders for these patients to avoid risk-taking behaviours. A key finding came from Haines et all [49], where results indicated that participants with impaired cognitive function in the complete programme (that included goal-setting and review) incurred a significantly higher rate of injurious falls per 1000 patient-days than those in the control group (7.49 vs. 2.89, p = 0.02). The authors stated that cognitive impairment may have constrained patients’ ability to adhere to safety plans, and that the education process may have made them more willing to report injuries from falls.

In a study of nurses’ experience of falls prevention, participants described using a combination of formal assessment, monitoring and communication as part of an ongoing strategy of ‘knowing that the patient is safe’ [56]. These strategies enabled nurses to be responsive to patients’ requests for help and ensure safety even if patients are unable to participate in interventions fully in response to messaging e.g., due to cognitive impairment. However, constraints were described for each strategy. For example, low staffing levels reduced nurse vigilance when making patient rounds and constrained the direct patient contact needed to know patients were safe.

#### Programme theory refinement

The Patient Participation analysis was used to refine the CMOcs and overarching IPT and summary statements reflecting refinements were assessed using GRADE-CerQual (see Table 4).

**Table 4:** Patient Participation Programme Theory Refinement

Context	Mechanism		Outcome	GRADE-CerQual assessment of confidence in summary statement.
	Resource	Response		
Patients with capacity have different perspectives and circumstances that may influence if/how they participate in falls prevention strategies in hospital. Staff have the time and skills to create an interactional rather than task focused space for assessment and care planning.	Staff individualise falls prevention messages for patients, i.e., that account for their circumstances and perspectives.	**Patient empowerment**: patients are empowered (increased confidence to ask for help, knowledge about their falls prevention strategy, acceptance of their falls risks) to participate in appropriate strategies.	Patients participate in interventions such as using the call bell and avoid risk taking behaviour.	**High confidence in Context and Mechanism summary statements**: It is highly likely that the review finding is a reasonable representation of the phenomena of interest. **Outcome**: limited data about the extent to which patients participate in interventions.
If staff are not responsive to patients’ requests for help mobilising or performing functional tasks e.g., due to task load / awareness.	Staff individualise goal setting and falls prevention messages to patient, i.e., that account for their circumstances and perspectives.	**Taking risks**: Patient confident they can, or feel urgency to, mobilise by themselves e.g., to get to the toilet.	Patient at risk of falling, particularly if hurrying.	**High confidence**: It is highly likely that the review finding is a reasonable representation of the phenomena of interest.
Patients with cognitive impairment have falls risk factors like other patients but additionally, depending on the severity of their cognitive impairment, may have problems with memory, attention and confusion.	Staff individualise falls prevention messages to patient i.e., that address their emotional barriers to participation.	**Taking risks**: Patients may struggle to understand or retain information and are unable to communicate needs unambiguously to staff, despite messaging.	Patients engage in risk taking behaviour.	**High confidence in Context and Outcome**: It is highly likely that the review finding is a reasonable representation of the phenomena of interest. **Mechanism**: limited data about how patients with cognitive impairment respond to interventions designed to encourage their participation.
Patients with cognitive impairment have falls risk factors like other patients but additionally may have problems with memory, attention and confusion.	Staff undertake ongoing assessment of risk and monitoring of patient.	**Knowing the patient is safe**: Staff collate the information necessary to understand if the patient is safe from harm.	Staff intervene in behaviour that may lead to a fall.	**Not assessed** due to paucity of data about patients with cognitive impairment.

## Discussion

This study aimed to explore why there is variation in implementation of MFRAs and interventions tailored to address individual falls risk factors in acute hospitals and focused on the role of MFRA tools and patient participation.

MFRA tools provide a structure for assessing patient falls risk factors. The analysis suggested that if tools are visible in staff workflow, they can facilitate delivery of falls prevention practices by prompting assessment of falls risk factors and identification of appropriate interventions [15, 26, 29, 31, 33]. Additionally, HIT appeared a promising implementation support, automating some practices for clinical staff [20, 23, 25]. Several factors helped explain variation in practices as documented in clinical records; tools differ in the number and type of items included, and in terms of whether they stratify (and standardise interventions) by risk - a practice no longer recommended in NICE guidance. Tool use may be disrupted where stratification by risk does not align with clinical judgement [31, 42] and because hospital wards are complex environments: patients’ condition changes; they may move between beds and wards; different professional groups are involved in intervention delivery; and there is variation in availability of falls prevention interventions [21, 32–34, 38, 40]. These interacting factors influence the extent to which falls prevention practices are enacted and documented as intended.

The literature highlighted the important role that patients can play in falls prevention, an area that has received less attention than other types of intervention [66]. Analysis suggested that patient-directed messaging is more likely to lead to their participation in interventions where individual circumstances and perspectives are considered, e.g., not wanting to disturb busy nurses by using the call bell [53, 61–63]. The quality of interaction between patients and professionals appeared to underpin successful messaging [53, 54], enabling patients to express the circumstances that constrained their participation. However, creating this interactional space appeared to rely on staff experience, skills, and time – resources that may be limited outside a study context [53, 54]. Furthermore, some patients are not able to remember or understand messaging due to cognitive impairment, with one study indicating potential harm to these patients [49]. Therefore, other strategies are needed to support falls prevention in these populations.

### Strengths and limitations

Theory development was achieved *via* iterative searches of the literature, building on practitioner ideas with evidence from empirical studies, and allowed for the inclusion of different types of data. Including different methodologies was considered a study strength, providing examples from clinical practice in the form of quality improvement projects. However, data synthesis was challenging because there was much variation e.g., in outcomes assessed, description of methods, and the data reported. Additionally, data evidencing staff experiences of MFRA tool use was limited, impacting quality assessment of the key findings using GRADE-CerQual, see Appendix iv. Facilitation *via* tools and Patient Participation were prioritised for exploration in this review but further work is needed to build explanation about the delivery of interventions to modify individual risk factors post-MFRA e.g., how information and action is shared between multidisciplinary teams to ensure each risk factor is addressed for patients.

## Conclusions

Implementation of multifactorial falls risk assessment and tailored interventions is supported where tools are visible to staff in their workflow to prompt practice. There is variation in falls prevention practices partly because the content of MFRA tools differs across organisations and because ward conditions, such as bed swaps and the availability of interventions, influence the extent to which practices are enacted. Patient-directed messaging that accounts for their personal circumstances, such as reluctance to disturb busy nurses, is more likely to lead to patient participation in interventions but creating interactional spaces that elicit these circumstances can be resource intensive. Furthermore, some patients, e.g., those with cognitive impairment, may not be able to participate despite appropriately directed messages.

## Electronic supplementary material

Below is the link to the electronic supplementary material.

Supplementary Material 1

Supplementary Material 2

Supplementary Material 3

Supplementary Material 4

## Data Availability

Data supporting the findings of this study are available in published studies referenced within the article.
