# Supplementary material for: Exploring variation in implementation of multifactorial falls risk assessment and tailored interventions: a realist review
Source: BMC Geriatr. 2023 Jun 21;23:381. doi: 10.1186/s12877-023-04045-3 (PMC10286425; doi:10.1186/s12877-023-04045-3)
Supplement: Supplementary file 1 — Supplementary Material 1 [file 12877_2023_4045_MOESM1_ESM.docx]

Appendix 1: Stage 1 Searches: Theory Construction

This appendix reports the final search strategies used for all databases searched for stage 1. On 22nd July 2020 we conducted three sets of searches, each comprising several database searches. The purpose of the search was to identify literature describing assumptions and theories underlying fall risk assessments in acute hospital settings. Subject headings and free text words were identified for use in the search concepts for all searches by the Information Specialist and project team members. The searches were peer-reviewed by an Information Specialist.

The table below summarises the databases searched and the order of search strategies presented below the table.

Table X. Stage 1 literature search sources searched and publication types sought.

| **Search name** | **Databases searched** | **Type of publication or study** |
| --- | --- | --- |
| 1.1 Practitioner Theory Search | CINAHL (EBSCOhost)  HMIC Health Management Information Consortium (Ovid)  Ovid MEDLINE(R) and Epub Ahead of Print, In-Process & Other Non-Indexed Citations and Daily <1946 to July 21, 2020> | Commentary pieces  Policy documents Studies mentioning theories or theoretical concepts |
| 1.2 Key Journal search | CINAHL (EBSCOhost)  Embase Classic+Embase (Ovid) <1947 to 2020 July 21>  HMIC Health Management Information Consortium (Ovid)  Ovid MEDLINE(R) and Epub Ahead of Print, In-Process & Other Non-Indexed Citations and Daily <1946 to July 21, 2020> | Trade journal article or news item |
| 1.3 Academic theory search | CINAHL (EBSCOhost)  Cochrane Central Register of Controlled Trials (Wiley) Issue 7 of 12, July 2020  Epistemonikos <https://www.epistemonikos.org/>  HMIC Health Management Information Consortium (Ovid)  International HTA Database (INAHTA) <https://database.inahta.org/>  Ovid MEDLINE(R) and Epub Ahead of Print, In-Process & Other Non-Indexed Citations and Daily <1946 to July 21, 2020>  PROSPERO <https://www.crd.york.ac.uk/prospero/> | Systematic Review |

Practitioner Theory Searches

**CINAHL (EBSCOhost) 1981- present**

**Date Run: 22/07/2020**

S29 S22 AND S28 220

S28 S23 OR S24 OR S25 OR S26 OR S27 443,776

S27 TI ( (view or views) ) OR TI editorial OR TI letter* 88,866

S26 TI "Comment on" OR TI commentary OR TI opinion* 60,866

S25 (MH "News") 9,948

S24 (MH "Policy and Procedure Manuals") OR (MH "Hospital Policies") OR (MH "Practice Guidelines") OR (MH "Health Policy") OR (MH "Public Policy") 156,362

S23 TI ( (policy or policies or guideline* or recommendation* or position) ) OR TI ( (theor* or concep* or logic) ) OR AB ( (theor* or concep* or logic) n1 (framework* or model* or analy* or evaluat*) ) 183,949

S22 S12 AND S21 3,341

S21 S13 OR S14 OR S15 OR S16 OR S17 OR S18 OR S19 OR S20 588,974

S20 TI hospital* OR AB hospital* 443,811

S19 TI ( inpatient or inpatients) OR AB ( inpatient or inpatients) 50,179

S18 TI ( ((rehabilitation or geriatric) n1 (ward? or unit? or department?)) ) OR AB ( ((rehabilitation or geriatric) n1 (ward? or unit? or department?)) ) 4,564

S17 TI ( ((acute or sub-acute or subacute) n3 (care or ward?)) ) OR AB ( ((acute or sub-acute or subacute) n3 (care or ward?)) ) 25,578

S16 (MH "Inpatients") 81,879

S15 (MH "Hospital Units+") 95,192

S14 (MH "Subacute Care") OR (MH "Acute Care") 9,672

S13 (MH "Hospitalization") 35,680

S12 S10 OR S11 11,240

S11 (MH "Morse Fall Scale") OR (MH "Fall Risk Assessment Tool") OR (MH "Fall Risk (Saba CCC)") OR (MH "Fall Prevention (Iowa NIC)") OR (MH "Hendrich Fall Risk Model") OR (MH "Safety Behavior: Fall Prevention (Iowa NOC)") 380

S10 S4 AND S9 11,097

S9 S5 OR S6 OR S7 OR S8 200,311

S8 (MH "Patient Safety") 60,372

S7 TI ( (fall* n3 (assess* or screen* or prevent* or predict*)) ) OR AB ( (fall* n3 (assess* or screen* or prevent* or predict*)) ) 8,089

S6 TI risk assess* OR AB risk assess* 47,861

S5 (MH "Risk Assessment") 103,677

S4 S1 OR S2 OR S3 50,072

S3 TI ( fall or falls or faller* ) OR AB ( fall or falls or faller* ) 41,406

S2 (MH "Hip Fractures/PC") 864

S1 (MH "Accidental Falls") 22,724

**HMIC Health Management Information Consortium (Ovid) <1983 - present>**

**Date Run: 22/07/2020**

1 falling/ (563)

2 (fall or falls or faller*).tw. (2653)

3 1 or 2 (2745)

4 exp risk assessment/ or risk management/ or risks/ (5389)

5 risk assess*.tw. (1136)

6 (fall* adj3 (assess* or screen* or prevent* or predict*)).tw. (383)

7 accident prevention/ or preventive measures/ or safety measures/ (6726)

8 4 or 5 or 6 or 7 (12379)

9 Commentaries/ (18)

10 "Comment on".ti. (29)

11 editorial.ti. (279)

12 letter.ti. (398)

13 Opinions/ (778)

14 opinion*.ti. (516)

15 Views/ (6902)

16 (view or views).ti. (3583)

17 (letter* adj3 editor*).ti. (1)

18 (evidence* adj8 (policy or policies)).tw. (1441)

19 policy/ or health policy/ or public policy/ (9125)

20 (policy or policies or guideline* or recommendation* or position).ti. (12388)

21 (theor* or concep* or logic).ti. (2829)

22 ((theor* or concep* or logic) adj (framework* or model* or analy* or evaluat*)).ab. (1897)

23 or/9-22 [Theories] (33927)

24 3 and 8 and 23 [Falls AND Risks AND Theories] (30)

**Ovid MEDLINE(R) and Epub Ahead of Print, In-Process & Other Non-Indexed Citations and Daily <1946 to July 21, 2020>**

**Date Run: 22/07/2020**

1 Accidental Falls/ or exp Hip Fractures/pc (25500)

2 (fall or falls or faller*).tw,kw. (147448)

3 or/1-2 [falls] (156010)

4 Risk Assessment/ (265251)

5 risk assess*.tw,kw. (69315)

6 (fall* adj3 (assess* or screen* or prevent* or predict*)).tw,kw. (10733)

7 exp Accident Prevention/ (86806)

8 or/4-7 [assessment or prevention] (391875)

9 Hospitalization/ (107412)

10 Subacute Care/ (1049)

11 Hospital Units/ (10146)

12 exp Hospitals/ (274581)

13 Rehabilitation Centers/ (8183)

14 Inpatients/ (21949)

15 ((acute or sub-acute or subacute) adj3 (care or ward?)).tw,kw. (31737)

16 ((rehabilitation or geriatric) adj (ward? or unit? or department?)).tw,kw. (6423)

17 inpatient?.tw,kw. (107879)

18 hospital*.tw,kw. (1294309)

19 or/9-18 [hospital] (1485504)

20 3 and 8 and 19 [Fall assmt & prevention in hospitals] (3313)

21 (policy or policies or guideline* or recommendation* or position).ti. (209315)

22 guideline/ or practice guideline/ (34299)

23 policy/ or public policy/ or exp health policy/ (138961)

24 (theor* or concep* or logic).ti. (206285)

25 ((theor* or concep* or logic) adj (framework* or model* or analy* or evaluat*)).ab. (74749)

26 or/21-25 [Policy, Guideline or overt Theory] (599087)

27 Comment/ (861900)

28 Letter/ (1091147)

29 Editorial/ (536220)

30 news/ or newspaper article/ (218702)

31 "Comment on".ti. (27827)

32 (letter* adj3 editor*).ti. (17425)

33 opinion*.ti. (15543)

34 (view or views).ti. (54684)

35 or/27-34 [Discussion papers Hidden Theory] (2145770)

36 26 or 35 [Theory Search] (2683132)

37 3 and 8 and 19 and 36 (177)

Key Journal Search

Relevant falls risk assessment articles and commentaries were search for in the following key trade magazines or journals: Nursing Standard, Nursing Times, Pharmaceutical Journal, Health Service Journal. Databases covering these key journals were searched.

**CINAHL (EBSCOhost) 1981- present**

**Date Run: 22/07/2020**

**# Query Results**

S17 S14 OR S16 168

S16 S12 AND S15 79

S15 JN nursing standard 59,378

S14 S12 AND S13 89

S13 JN nursing times 52,673

S12 S10 OR S11 11,240

S11 S4 AND S9 11,097

S10 (MH "Morse Fall Scale") OR (MH "Fall Risk Assessment Tool") OR (MH "Fall Risk (Saba CCC)") OR (MH "Fall Prevention (Iowa NIC)") OR (MH "Hendrich Fall Risk Model") OR (MH "Safety Behavior: Fall Prevention (Iowa NOC)") 380

S9 S5 or S6 or S7 or S8 200,311

S8 (MH "Patient Safety") 60,372

S7 TI ( (fall* n3 (assess* or screen* or prevent* or predict*)) ) OR AB ( (fall* n3 (assess* or screen* or prevent* or predict*)) ) 8,089

S6 TI risk assess* OR AB risk assess* 47,861

S5 (MH "Risk Assessment") 103,677

S4 S1 OR S2 or S3 50,072

S3 TI ( fall or falls or faller* ) OR AB ( fall or falls or faller* ) 41,406

S2 (MH "Hip Fractures/PC") 864

S1 (MH "Accidental Falls") 22,724

**Embase Classic+Embase (Ovid) <1947 to 2020 July 21>**

**Date Run: 22/07/2020**

1 (fall or falls or faller*).tw. (220189)

2 falling/ (40451)

3 1 or 2 (237166)

4 risk assessment/ (567038)

5 risk assess*.tw,kw. (97678)

6 (fall* adj3 (assess* or screen* or prevent* or predict*)).tw,kw. (15117)

7 accident prevention/ (17289)

8 hip fracture/pc [Prevention] (2210)

9 4 or 5 or 6 or 7 or 8 (622377)

10 3 and 9 (21538)

11 pharmaceutical journal.jn. (21130)

12 10 and 11 (10)

**HMIC Health Management Information Consortium (Ovid) <1983 - present>**

**Date Run: 22/07/2020**

1 falling/ (563)

2 (fall or falls or faller*).tw. (2653)

3 1 or 2 (2745)

4 exp risk assessment/ or risk management/ or risks/ (5389)

5 risk assess*.tw. (1136)

6 (fall* adj3 (assess* or screen* or prevent* or predict*)).tw. (383)

7 accident prevention/ or preventive measures/ or safety measures/ (6726)

8 4 or 5 or 6 or 7 (12379)

9 health service* journal.jn. (13050)

10 3 and 8 and 9 (10)

11 pharmaceutical journal.jn. (2407)

12 3 and 8 and 11 (4)

13 10 or 12 (14)

**Ovid MEDLINE(R) and Epub Ahead of Print, In-Process & Other Non-Indexed Citations and Daily <1946 to July 22, 2020>**

Date Run: 22/07/2020

1 Accidental Falls/ or exp Hip Fractures/pc (25502)

2 (fall or falls or faller*).tw,kw. (147405)

3 or/1-2 [falls] (155967)

4 Risk Assessment/ (265290)

5 risk assess*.tw,kw. (69279)

6 (fall* adj3 (assess* or screen* or prevent* or predict*)).tw,kw. (10727)

7 exp Accident Prevention/ (86820)

8 or/4-7 [assessment or prevention] (391878)

9 nursing time*.jn. (39139)

10 3 and 8 and 9 (26)

11 nursing standard.jn. (35276)

12 3 and 8 and 11 (33)

13 health service* journal.jn. (10933)

14 3 and 8 and 13 (3)

15 10 or 12 or 14 (62)

Academic Theory Search

**CINAHL (EBSCOhost) 1981- present**

**Date Run: 22/07/2020**

**# Query Results**

S25 S12 AND S21 AND S24 166

S24 S22 OR S23 188,189

S23 TI ( Literature review* or systematic n2 review* or narrative n2 review* or critical n2 review* or scoping review* or synthesis or meta-analys* or "meta analysis" or realist n2 review* ) OR AB ( "Search filter*" or "search strateg*" or "literature search*" ) 149,311

S22 (MH "Systematic Review") OR (MH "Literature Review") OR (MH "Scoping Review") OR (MH "Concept Analysis") 94,643

S21 S13 OR S14 OR S15 OR S16 OR S17 OR S18 OR S19 OR S20 588,974

S20 (MH "Inpatients") 81,879

S19 (MH "Hospital Units+") 95,192

S18 (MH "Subacute Care") OR (MH "Acute Care") 9,672

S17 (MH "Hospitalization") 35,680

S16 TI hospital* OR AB hospital* 443,811

S15 TI ( inpatient or inpatients) OR AB ( inpatient or inpatients) 50,179

S14 TI ( ((rehabilitation or geriatric) n1 (ward? or unit? or department?)) ) OR AB ( ((rehabilitation or geriatric) n1 (ward? or unit? or department?)) ) 4,564

S13 TI ( ((acute or sub-acute or subacute) n3 (care or ward?)) ) OR AB ( ((acute or sub-acute or subacute) n3 (care or ward?)) ) 25,578

S12 S10 OR S11 11,240

S11 (MH "Morse Fall Scale") OR (MH "Fall Risk Assessment Tool") OR (MH "Fall Risk (Saba CCC)") OR (MH "Fall Prevention (Iowa NIC)") OR (MH "Hendrich Fall Risk Model") OR (MH "Safety Behavior: Fall Prevention (Iowa NOC)") 380

S10 S4 AND S9 11,097

S9 S5 OR S6 OR S7 OR S8 200,311

S8 (MH "Patient Safety") 60,372

S7 TI ( (fall* n3 (assess* or screen* or prevent* or predict*)) ) OR AB ( (fall* n3 (assess* or screen* or prevent* or predict*)) ) 8,089

S6 TI risk assess* OR AB risk assess* 47,861

S5 (MH "Risk Assessment") 103,677

S4 S1 OR S2 OR S3 50,072

S3 TI ( fall or falls or faller* ) OR AB ( fall or falls or faller* ) 41,406

S2 (MH "Hip Fractures/PC") 864

S1 (MH "Accidental Falls") 22,724

**Cochrane Central Register of Controlled Trials (Wiley) Issue 7 of 12, July 2020**

**Date Run: 22/07/2020**

ID Search Hits

#1 MeSH descriptor: [Accidental Falls] this term only 1447

#2 MeSH descriptor: [Hip Fractures] explode all trees and with qualifier(s): [prevention & control - PC] 149

#3 (fall or falls or faller*):ti,ab,kw 17295

#4 #1 or #2 or #3 17383

#5 MeSH descriptor: [Risk Assessment] this term only 8650

#6 (risk assess*):ti,ab,kw 100689

#7 (fall* near/3 (assess* or screen* or prevent* or predict*)):ti,ab,kw 2943

#8 MeSH descriptor: [Accident Prevention] explode all trees 3928

#9 #5 or #6 or #7 or #8 106001

#10 #4 and #9 4842

#11 MeSH descriptor: [Hospitalization] this term only 5065

#12 MeSH descriptor: [Subacute Care] this term only 16

#13 MeSH descriptor: [Hospital Units] this term only 194

#14 MeSH descriptor: [Hospitals] explode all trees 3555

#15 MeSH descriptor: [Rehabilitation Centers] this term only 308

#16 MeSH descriptor: [Inpatients] this term only 924

#17 ((acute or sub-acute or subacute) near/3 (care or ward?)):ti,ab,kw 3091

#18 ((rehabilitation or geriatric) next (ward? or unit? or department?)):ti,ab,kw 1226

#19 inpatient*:ti,ab,kw 17486

#20 hospital*:ti,ab,kw 168547

#21 ^160-#20^ 177578

#22 #4 and #9 and #21 1141

(CDSR = 114)

**Epistemonikos (Epistemonikos Foundation) - all available dates**

**Date Run: 22/07/2020**

Title/Abstract: fall OR falls OR faller*

AND

Title/Abstract: "risk assessment" or "risk prevention" or "falls assessment" or "falls prevention"

AND

Title/Abstract: hospital* or inpatient* or ward or wards or acute

Limited: Systematic Reviews

49hits

**Database: HMIC Health Management Information Consortium (Ovid) <1983 - present>**

**Date Run: 22/07/2020**

1 falling/ (563)

2 (fall or falls or faller*).tw. (2653)

3 1 or 2 (2745)

4 exp risk assessment/ or risk management/ or risks/ (5389)

5 risk assess*.tw. (1136)

6 (fall* adj3 (assess* or screen* or prevent* or predict*)).tw. (383)

7 accident prevention/ or preventive measures/ or safety measures/ (6726)

8 4 or 5 or 6 or 7 (12379)

9 exp hospitals/ (15398)

10 ((acute or sub-acute or subacute) adj3 (care or ward?)).tw. (2677)

11 ((rehabilitation or geriatric) adj (ward? or unit? or department?)).tw. (524)

12 inpatient?.tw. (3739)

13 hospital*.tw. (49895)

14 or/9-13 [Acute Hosp Wards] (57296)

15 3 and 8 and 14 (197)

16 systematic reviews/ or literature reviews/ or meta analysis/ (6695)

17 (Literature review* or (systematic adj2 review*) or (narrative adj2 review*) or (critical adj2 review*) or scoping review* or synthesis or meta-analys* or "meta analysis" or (realist adj2 review*)).ti. (4659)

18 ("Search filter*" or "search strateg*" or "literature search*").ab. (1140)

19 or/16-18 [Systematic Reviews] (8438)

20 15 and 19 (13)

**Ovid MEDLINE(R) and Epub Ahead of Print, In-Process & Other Non-Indexed**

**Citations and Daily <1946 to July 21, 2020>**

**Date Run: 22/07/2020**

1 Accidental Falls/ or exp Hip Fractures/pc (25500)

2 (fall or falls or faller*).tw,kw. (147448)

3 or/1-2 [falls] (156010)

4 Risk Assessment/ (265251)

5 risk assess*.tw,kw. (69315)

6 (fall* adj3 (assess* or screen* or prevent* or predict*)).tw,kw. (10733)

7 exp Accident Prevention/ (86806)

8 or/4-7 [assessment or prevention] (391875)

9 Hospitalization/ (107412)

10 Subacute Care/ (1049)

11 Hospital Units/ (10146)

12 exp Hospitals/ (274581)

13 Rehabilitation Centers/ (8183)

14 Inpatients/ (21949)

15 ((acute or sub-acute or subacute) adj3 (care or ward?)).tw,kw. (31737)

16 ((rehabilitation or geriatric) adj (ward? or unit? or department?)).tw,kw. (6423)

17 inpatient?.tw,kw. (107879)

18 hospital*.tw,kw. (1294309)

19 or/9-18 [hospital] (1485504)

20 3 and 8 and 19 [Fall assmt & prevention in hospitals] (3313)

21 meta-analysis/ or "systematic review"/ (194072)

22 (Literature review* or (systematic adj2 review*) or (narrative adj2 review*) or (critical adj2 review*) or scoping review* or synthesis or meta-analys* or "meta analysis" or (realist adj2 review*)).ti. (551543)

23 ("Search filter*" or "search strateg*" or "literature search*").ab. (66610)

24 or/21-23 [Systematic reviews] (635237)

25 20 and 24 (139)

**PROSPERO International prospective register of systematic reviews (NIHR) - all available dates**

**Date Run: 22/07/2020**

Title only search: (falls or fall or faller*) and (risk or assess*)

62 hits

International HTA Database (INAHTA) – all available dates

Date Run: 23/07/2020

17 #16 AND #8 AND #7 21

16 #15 OR #14 OR #13 OR #12 OR #11 OR #10 OR #9 1791

15 (hospital* OR inpatient* or ward or wards or acute)[Title] OR (hospital* OR inpatient* or ward or wards or acute)[abs] 1760

14 "Inpatients"[mh] 42

13 "Rehabilitation Centers"[mh] 10

12 "Hospital Units"[mh] 7

11 "Subacute Care"[mh] 0

10 "Hospitals"[mhe] 64

9 "Hospitalization"[mh] 28

8 #6 OR #5 OR #4 OR #3 803

7 #2 OR #1 103

6 "Accident Prevention"[mhe] 97

5 (fall* AND (assess* or screen* or prevent* or predict*))[Title] OR (fall* AND (assess* or screen* or prevent* or predict*))[abs] 72

4 "Risk Assessment"[mh] 102

3 (risk assess*)[Title] OR (risk assess*)[abs] 609

2 (fall or falls or faller*)[Titl e] OR (fall or falls or faller*)[abs] 102

1 "Accidental Falls"[mh] 18
