# Supplementary material for: Exploring variation in implementation of multifactorial falls risk assessment and tailored interventions: a realist review
Source: BMC Geriatr. 2023 Jun 21;23:381. doi: 10.1186/s12877-023-04045-3 (PMC10286425; doi:10.1186/s12877-023-04045-3)
Supplement: Supplementary file 2 — Supplementary Material 2 [file 12877_2023_4045_MOESM2_ESM.docx]

Appendix 2: Programme Theory Testing Searches

This appendix reports the final search strategies used for all databases searched for stage 2.

- Search 2.1 Embase scoping search of 6 theories
- Search 2.2 Multiple database search of 4 theories
- Search 2.3 Update search of 2 prioritised theories

The purpose of these searches was to identify literature that could provide evidence to support, refute or refine our chosen theories. The first search scoped six potential CMO’s identified by the project team. This search was conducted in EMBASE only to gauge the size of the relevant literature in each of the six proposed CMOs and refine the search before translating into other databases.

The CMO list was refined into four search questions for search 2.2 and run in multiple databases (detailed below). The final search (2.3) updated the two theories (CMOs) that had been prioritised for full review.

Subject headings and free text words were identified for use in the search concepts for all searches by the Information Specialist and project team members. The searches were peer-reviewed by an Information Specialist.

The table below summarises the three searches and the order of search strategies presented below the table.

Table: Stage 2 literature search sources searched and scope of theories covered.

| **Search name and scope** | **Search date** | **Databases searched** |
| --- | --- | --- |
| 2.1  6 theories:  Leadership  Staff training /Empowerment  Assessment tools /Health Information Technologies  Patient centred care  Staff expertise  Shared responsibility / teamwork | 02-03-21 | Embase <1996 to 2021 Week 08> |
| 2.2  4 Theories:  Leadership  Facilitation  Patient Partnership  Shared Responsibility | 06-05-21 and  07-05-21 | CINAHL (EBSCOhost)  Embase Classic+Embase  (Ovid) 1947 to 2021 May 05  Ovid MEDLINE(R) ALL 1946 to May 05, 2021  Arts & Humanities Citation Index (Web of Science) 1975+  Conference Proceedings Citation Index- Science ( Web of Science) 1990+  Conference Proceedings Citation Index- Social Science & Humanities (Web of Science) 1990+  Science Citation Index-Expanded (Web of Science) 1900+  Social Sciences Citation Index (Web of Science) 1900+  Emerging Sources Citation Index (Web of Science) 2015+  NICE Evidence <https://www.evidence.nhs.uk/> |
| 2.3  2 Prioritised theories:  Facilitation  Patient Partnership | 01-08-22 | CINAHL (EBSCOhost)  Embase Classic+Embase (Ovid) 1947 to 2022 July 29  Ovid MEDLINE(R) ALL 1946 to July 29, 2022  Arts & Humanities Citation Index (Web of Science) 1975+  Conference Proceedings Citation Index- Science ( Web of Science) 1990+  Conference Proceedings Citation Index- Social Science & Humanities (Web of Science) 1990+  Science Citation Index-Expanded (Web of Science) 1900+  Social Sciences Citation Index (Web of Science) 1900+  Emerging Sources Citation Index (Web of Science) 2015+ |

Search 2.1: Embase scoping search of 6 theories

**Embase (Ovid) 1996 to 2021 Week 08**

**Search date: 02/03/2021**

Records found: 1358 in total from download of 6 search lines (lines 40,53, 64,76, 86, 96).  These were de-duplicated to leave 970 records in the EndNote Library

Search Strategy:

--------------------------------------------------------------------------------

1     hospitalization/ (380692)

2     hospital department/ or exp ward/ (394030)

3     exp hospital/ (1060104)

4     medical staff/ (30743)

5     nursing staff/ (53204)

6     rehabilitation center/ (13386)

7     subacute care/ (1105)

8     exp clinical handover/ or collaborative care team/ or exp rapid response team/ (10875)

9     exp hospital patient/ (184493)

10     ((acute or sub-acute or subacute) adj3 (care or ward?)).tw,kw. (45721)

11     ((rehabilitation or geriatric) adj (ward? or unit? or department?)).tw,kw. (10024)

12     inpatient?.tw,kw. (176834)

13     (acute adj5 patient?).tw,kw. (302957)

14     hospital*.tw,kw. (1829684)

15     or/1-14 [hospital] (2688257)

16     *falling/ (11365)

17     falling/pc (2719)

18     (fall? adj2 (assess* or risk? or prevent* or reduc* or occur* or frequenc* or screen)).tw,kw. (22530)

19     or/16-18 [Falls prevention or risk assessment] (28603)

20     implement*.tw,kw. (672165)

21     (sustain* adj4 (program* or practice* or intervention?)).tw,kw. (13916)

22     Adopt*.tw,kw. /freq=2 (44117)

23     Adopt*.ti. (16283)

24     deliver*.tw,kw. /freq=2 (336819)

25     (adher* or comply or complian*).tw,kw. (452974)

26     fidelity.tw,kw. (32350)

27     implementation science/ (1734)

28     exp protocol compliance/ (15146)

29     *health care planning/ (21195)

30     *practice guideline/ (77969)

31     or/20-30 [Implementation or Adherence to Guidelines and strategies] (1538880)

32     15 and 19 and 31 [Implementation AND Falls Prevention AND Hospitals] (1542)

33     leadership/ (67428)

34     total quality management/ (69290)

35     leader*.tw,kw. (86965)

36     champion*.tw,kw. (9647)

37     (organi#ation* adj3 (support* or strateg*)).tw,kw. (11289)

38     exp *"organization and management"/ (410957)

39     or/33-38 [Leadership] (558040)

40     32 and 39 [Leadership Falls Implementation Hospitals] (371)

41     staff training/ (13513)

42     continuing education/ (28691)

43     *medical education/ (75344)

44     *nursing education/ (29715)

45     (training* adj3 (staff or professional? or nurs* or doctor? or clinical or medical or clinician?)).tw,kw. (56356)

46     (educat* adj3 (staff or professional? or nurs* or doctor? or clinical or medical or clinician?)).tw,kw. (125827)

47     (empower* adj3 (staff or professional? or nurs* or doctor? or clinician?)).tw,kw. (2807)

48     (accountab* adj3 (staff or professional? or nurs* or doctor? or clinician?)).tw,kw. (976)

49     (responsib* adj3 (staff or professional? or nurs* or doctor? or clinician?)).tw,kw. (9080)

50     ((fedback* or feedback* or fed-back* or feed-back*) adj3 (staff or professional? or nurs* or doctor? or clinician?)).tw,kw. (3902)

51     empowerment/ (10465)

52     or/41-51 [Staff training and empowerment] (289563)

53     32 and 52 [Staff training Empowerment and Falls Implementation Hospitals] (182)

54     clinical assessment tool/ (24623)

55     (assess* adj4 tool?).tw,kw. (89900)

56     (electronic adj2 record?).tw,kw. (80127)

57     *fall risk assessment/ (588)

58     medical informatics/ or nursing informatics/ (21855)

59     exp hospital information system/ (20817)

60     exp information technology device/ (133716)

61     (information adj3 (technolog* or system or computeri* or electronic)).tw,kw. (62537)

62     digital*.tw,kw. (169019)

63     or/54-62 [Assessment tools or health info technology] (552944)

64     32 and 63 [Assessment Tools or HIT and Falls Implementation Hospitals] (261)

65     (fall* adj6 (decreas* or declin* or reduc* or lower* or fewer or less*)).tw,kw. [REDUCTION in falls] (17691)

66     patient care/ (282843)

67     shared medical appointment/ (126)

68     (patient? adj2 (centred or centered)).tw,kw. (35234)

69     (patient? adj5 perspective?).tw,kw. (31276)

70     (patient? adj4 need?).tw,kw. (109033)

71     patient participation/ (26089)

72     (engag* adj3 (patient? or famil* or carer? or caregiver? or player*)).tw,kw. (18827)

73     (multifacet* or multi-facet* or tailor*).tw,kw. (154544)

74     or/66-73 [Patient Centred Care] (604660)

75     15 and 19 and 74 [Patient Centred Care Falls Acute Hospital] (799)

76     15 and 19 and 65 and 74 [Patient Centred and Hospitals and Falls Reduction] (307)

77     medical expert/ (12634)

78     expert nurse/ (198)

79     nursing expertise/ (179)

80     expert*.tw,kw. (278203)

81     (experienced adj3 (staff or professional? or nurs* or doctor? or clinician? or physiotherapis? or pharmacist?)).tw,kw. (11376)

82     champion?.tw,kw. (6814)

83     (specialist? adj2 (fall? or nurse)).tw,kw. (7782)

84     or/77-83 [Expertise] (306887)

85     15 and 19 and 84 [Expertise and Falls Acute Hospital] (259)

86     15 and 19 and 65 and 84 [Expertise and Hospitals and Falls Reduction] (95)

87     multidisciplinary team/ or collaborative care team/ (11349)

88     ((share* or sharing) adj3 (responsibility or responsible or accountability or accountable)).tw,kw. (2621)

89     collaborat*.tw,kw. /freq=2 (46785)

90     (team* adj3 (share* or sharing or communic*)).tw,kw. (7307)

91     (team* adj3 (multidisciplin* or interdisciplin* or multi-disciplin* or inter-disciplin* or interprofessional* or inter-professional*)).tw,kw. (58028)

92     cooperation/ or teamwork/ (58992)

93     public relations/ (39835)

94     or/87-93 [Team collaboration] (192129)

95     15 and 19 and 94 [Teams and Falls Acute Hospital] (332)

96     15 and 19 and 65 and 94 [Teams and Hospitals and Falls Reduction] (142)

Search 2.2: Multiple database search of 4 theories

***Leadership***

**CINAHL (EBSCOhost)**

**Search Date: 06/05/2021**

**Records found: 122**

# Query Results

S31 S12 AND S15 AND S21 AND S25 AND S30 122

S30 S26 OR S27 OR S28 OR S29 245,878

S29 TI ( (risk n2 (assess* or evaluat*)) or guideline* or protocol* ) AND AB ( (risk n2 (assess* or evaluat*)) or guideline* or protocol* ) 33,732

S28 (MH "Protocols+") 42,099

S27 (MH "Practice Guidelines") 81,201

S26 (MH "Risk Assessment") 114,437

S25 S22 OR S23 OR S24 686,998

S24 TI ( leader* or champion* or facilitator* or (organi#ation* n3 (support* or strateg*)) or ((quality or safety or nurse* or matron) n3 lead*)) OR AB ( leader* or champion* or facilitator* or (organi#ation* n3 (support* or strateg*)) or ((quality or safety or nurse* or matron*) n3 lead*)) 96,386

S23 (MH "Quality Management, Organizational") OR (MM "Management+") 603,390

S22 (MH "Leadership") 44,897

S21 S16 OR S17 OR S18 OR S19 OR S20 384,391

S20 (MH "Guideline Adherence") 15,898

S19 (MH "Systems Implementation") OR (MH "Program Implementation") OR (MH "Implementation Science") 30,900

S18 TI ( engage* or "buy in" or (Cognitive n2 participat*) ) OR AB ("buy in" or (Cognitive n2 participat*) ) 16,158

S17 TI ( sustain* n4 (program* or practice or practices or intervention or interventions) ) OR AB ( sustain* n4 (program* or practice or practices or intervention or interventions) ) 6,822

S16 TI ( implement* or adopt* or deliver* or adher* or comply or complian* or fidelity ) OR AB ( implement* or adher* or comply or complian* or fidelity) 344,120

S15 S13 OR S14 18,729

S14 TI (fall* n2 (assess* or risk* or prevent* or reduc* or occur* or frequen* or screen*)) OR AB (fall* n2 (assess* or risk* or prevent* or reduc* or occur* or frequen* or screen*)) 14,235

S13 (MH "Accidental Falls/PC") 10,323

S12 S1 OR S2 OR S3 OR S4 OR S5 OR S6 OR S7 OR S8 OR S9 OR S10 OR S11 829,664

S11 (MH "Multidisciplinary Care Team+") 46,811

S10 (MH "Medical Staff, Hospital+") OR (MH "Nursing Staff, Hospital") 27,035

S9 (MH "Hospitals+") 121,072

S8 (MH "Hospital Units") OR (MH "Rehabilitation Centers") 15,793

S7 (MH "Inpatients") 83,955

S6 (MH "Subacute Care") OR (MH "Acute Care") 10,376

S5 (MH "Hospitalization") 38,763

S4 TI hospital* OR AB hospital* 476,803

S3 TI ( inpatient or inpatients) OR AB ( inpatient or inpatients) 54,348

S2 TI ( ((rehabilitation or geriatric) n1 (ward? or unit? or department?)) ) OR AB ( ((rehabilitation or geriatric) n1 (ward? or unit? or department?)) ) 4,834

S1 TI ( ((acute or sub-acute or subacute) n3 (care or ward?)) ) OR AB ( ((acute or sub-acute or subacute or patient*) n3 (care or ward? or patient*)) ) 258,661

**Embase Classic+Embase  (Ovid) 1947 to 2021 May 05**

**Search Date: 06/05/2021**

**Records found: 187**

--------------------------------------------------------------------------------

1     hospitalization/ (420992)

2     hospital department/ or exp ward/ (439715)

3     exp hospital/ (1302934)

4     medical staff/ (40310)

5     nursing staff/ (74427)

6     rehabilitation center/ (17387)

7     subacute care/ (1151)

8     exp clinical handover/ or collaborative care team/ or exp rapid response team/ (11414)

9     exp hospital patient/ (199241)

10     ((acute or sub-acute or subacute) adj3 (care or ward?)).tw,kw. (51102)

11     ((rehabilitation or geriatric) adj (ward? or unit? or department?)).tw,kw. (12324)

12     inpatient?.tw,kw. (199814)

13     (acute adj5 patient?).tw,kw. (369927)

14     hospital*.tw,kw. (2200719)

15     or/1-14 [hospital] (3282770)

16     *falling/ (12578)

17     falling/pc (2954)

18     (fall? adj2 (assess* or risk? or prevent* or reduc* or occur* or frequen* or screen*)).tw,kw. (25940)

19     or/16-18 [Falls prevention or risk assessment] (32853)

20     implement*.tw,kw. (724768)

21     (sustain* adj4 (program* or practice* or intervention?)).tw,kw. (14660)

22     Adopt*.tw,kw. /freq=2 (49567)

23     Adopt*.ti. (20665)

24     deliver*.tw,kw. /freq=2 (374349)

25     deliver*.ti. (176933)

26     (adher* or comply or complian*).tw,kw. (526988)

27     fidelity.tw,kw. (35433)

28     implementation science/ (1982)

29     exp protocol compliance/ (15723)

30     engage*.ti. (20887)

31     engage*.tw,kw. /freq=2 (52149)

32     (intervention adj2 deliver*).tw,kw. (7304)

33     (Cognitive adj2 participat*).tw,kw. (729)

34     "buy in".tw,kw. (2788)

35     or/20-34 [Implementation or Adherence to Guidelines and strategies] (1730025)

36     leadership/ (76777)

37     total quality management/ (72191)

38     leader*.tw,kw. (102125)

39     champion*.tw,kw. (10691)

40     (organi#ation* adj3 (support* or strateg*)).tw,kw. (12500)

41     exp *"organization and management"/ (577268)

42     facilitator*.ti. (6305)

43     facilitat*.tw,kw. /freq=2 (94889)

44     ((quality or safety or nurse* or matron*) adj3 lead*).tw,kw. (13991)

45     or/36-44 [Leaderships] (841009)

46     risk assessment/ (618392)

47     (risk adj2 (assess* or evaluat*)).tw,kw. (196115)

48     exp practice guideline/ (601575)

49     guideline*.tw,kw. (613857)

50     exp clinical protocol/ (106751)

51     protocol?.tw,kw. /freq=2 (185559)

52     protocol?.ti. (86947)

53     or/46-52 [Multifactorial risk assessment] (1767880)

54     15 and 19 and 35 and 45 and 53 [Leadership CMO1 - final] (187)

**Ovid MEDLINE(R) ALL 1946 to May 05, 2021**

**Search Date: 06/05/2021**

**Records found: 145**

 1     Hospitalization/ (115592)

2     Subacute Care/ (1169)

3     Hospital Units/ (10319)

4     exp Hospitals/ (284007)

5     medical staff, hospital/ or nursing staff, hospital/ (66506)

6     exp Patient Care Team/ (70042)

7     Rehabilitation Centers/ (8356)

8     Inpatients/ (23637)

9     ((acute or sub-acute or subacute) adj3 (care or ward?)).tw,kf. (34662)

10     ((rehabilitation or geriatric) adj (ward? or unit? or department?)).tw,kf. (6803)

11     (acute adj5 patient?).tw,kf. (227174)

12     inpatient?.tw,kf. (116838)

13     hospital*.tw,kf. (1383898)

14     or/1-13 [hospital] (1835443)

15     Accidental Falls/pc [Prevention & Control] (9549)

16     (fall? adj2 (assess* or risk? or prevent* or reduc* or occur* or frequen* or screen*)).tw,kf. (17142)

17     15 or 16 [Falls] (21139)

18     implement*.tw,kf. (545160)

19     (sustain* adj4 (program* or practice* or intervention?)).tw,kf. (11220)

20     Adopt*.tw,kf. /freq=2 (37248)

21     Adopt*.ti. (16394)

22     deliver*.tw,kf. /freq=2 (258583)

23     deliver*.ti. (137239)

24     (adher* or comply or complian*).tw,kf. (343916)

25     fidelity.tw,kf. (30445)

26     Health Plan Implementation/ (6379)

27     implementation science/ or technology transfer/ (2784)

28     Guideline Adherence/ (33374)

29     engage*.tw,kf. /freq=2 (39474)

30     engage*.ti. (17196)

31     (intervention adj2 deliver*).tw,kf. (5693)

32     (Cognitive adj2 participat*).tw,kf. (547)

33     "buy in".tw,kf. (1792)

34     or/18-33 [Implementation or Adherence to Guidelines and strategies] (1263268)

35     Leadership/ (42613)

36     Total Quality Management/ (12588)

37     exp *"organization and administration"/ (729180)

38     leader*.tw,kf. (83493)

39     champion*.tw,kf. (7722)

40     (organi#ation* adj3 (support* or strateg*)).tw,kf. (10007)

41     facilitator*.ti. (5290)

42     facilitat*.tw,kf. /freq=2 (72113)

43     ((quality or safety or nurse* or matron*) adj3 lead*).tw,kf. (10657)

44     35 or 36 or 37 or 38 or 39 or 40 or 41 or 42 or 43 [Leadership] (899951)

45     exp Risk Assessment/ (284216)

46     (risk adj2 (assess* or evaluat*)).tw,kf. (137873)

47     guideline/ or practice guideline/ (35660)

48     guideline*.tw,kf. (386469)

49     protocol?.tw,kf. /freq=2 (126031)

50     protocol?.ti. (69596)

51     exp Clinical Protocols/ (174448)

52     or/45-51 [Multifactorial risk assessment] (1055597)

53     14 and 17 and 34 and 44 and 52 [Leadership CMO1 final] (145)

**NICE Evidence**

**Search Date: 07/05/2021**

**Records found: 53**

("falls prevention" or "falls assessment" or "falls reduction") and (hospital or inpatient or "acute care" or "acute ward" or "acute patient" or "rehabilitation unit" or "rehabilitation ward" ) and (leadership or champions) and ("risk assessment" or "risk evaluation" or "evaluation of risk")

Sorted by Relevance .| Sort by Date

Filters applied:

Evidence type : Evidence Summaries

Evidence type: Primary Research

Evidence type: Systematic Reviews

Evidence type: Audit and Inspection Reports

Evidence type: Health Technology Assessments

**Web of Science Core Collection Databases available at the University of Leeds (searched simultaneously):**

Arts & Humanities Citation Index (Web of Science) 1975-present

Conference Proceedings Citation Index- Science ( Web of Science) 1990-present

Conference Proceedings Citation Index- Social Science & Humanities (Web of Science) 1990-present

Science Citation Index-Expanded (Web of Science) 1900-present

Social Sciences Citation Index (Web of Science) 1900-present

Emerging Sources Citation Index (Web of Science) 2015-present

**Search Date: 06/05/2021**

**Records found: 38**

Indexes=SCI-EXPANDED, SSCI, A&HCI, CPCI-S, CPCI-SSH, ESCI Timespan=1900-2021

# 18 38 #17 AND #14 AND #10 AND #6 AND #5

# 17 1,540,383 #16 OR #15

# 16 1,297,929 TOPIC: (guideline* or protocol*)

# 15 267,027 TOPIC: (risk near/2 (assess* or evaluat*) )

# 14 327,496 #13 OR #12 OR #11

# 13 22,680 TS=((quality or safety or nurse* or matron*) near/3 lead*)

# 12 37,727 TOPIC: (organi?ation* near/3 (support* or strateg*) )

# 11 279,723 TOPIC: (leader* or champion* or facilitator*)

# 10 2,790,544 #9 OR #8 OR #7

# 9 30,806 TOPIC: (sustain* near/4 (program* or practice* or intervention$) )

# 8 296,045 TI=(adopt* or deliver* or engage*)

# 7 2,495,757 TS=(implement* or adher* or comply or complian* or fidelity or "buy in" or (Cognitive near/2 participat*) )

# 6 28,251 TS=(fall$ near/2 (assess* or risk$ or prevent* or reduc* or occur* or frequen* or screen*) )

# 5 1,439,343 #4 OR #3 OR #2 OR #1

# 4 225,361 TOPIC: (acute near/5 patient$)

# 3 7,518 TOPIC: ((rehabilitation or geriatric) near/1 (ward$ or unit$ or department$) )

# 2 35,941 TOPIC: ((acute or sub-acute or subacute) near/3 (care or ward$) )

# 1 1,254,128 TOPIC: (hospital* OR inpatient*)

***Facilitation/Tools***

**CINAHL (EBSCOhost)**

**Search Date: 06/05/2021**

**Records found: 32**

S39 S12 AND S15 AND S21 AND S34 AND S38 32

S38 S35 OR S36 OR S37 260,284

S37 TI ( (facilitation or workflow* or work-flow* or embed* or integrat* or routine* or routini* or "system* fit*") ) OR AB ( (facilitation or workflow* or work-flow* or embed* or integrat* or routine* or routini* or "system* fit*") ) 252,176

S36 (MH "Health Care Delivery, Integrated") 12,531

S35 (MH "Systems Integration") OR (MH "Workflow") 4,610

S34 S22 OR S23 OR S24 OR S25 OR S26 OR S27 OR S28 OR S29 OR S30 OR S31 OR S32 OR S33 423,968

S33 (MH "Information Technology+") 18,859

S32 (MH "Medical Informatics") OR (MH "Nursing Informatics") OR (MH "Health Informatics") 12,075

S31 (MH "Clinical Information Systems+") OR (MH "Health Information Systems+") OR (MH "Hospital Information Systems") OR (MH "Nursing Information Systems+") OR (MH "Patient Record Systems+") OR (MH "Health Information Networks") OR (MH "Decision Support Systems, Clinical") 59,097

S30 TI "clinic* decision* support system*" OR AB "clinic* decision* support system*" 1,098

S29 TI (electronic* n3 nurs* n3 document*) OR AB (electronic* n3 nurs* n3 document*) 182

S28 TI (risk* n3 screen* n3 tool*) OR AB (risk* n3 screen* n3 tool*) 674

S27 TI ( ("risk assess*" n4 (bundle* or instrument* or care plan* or multi-factorial or multifactorial)) ) OR AB ( ("risk assess*" n4 (bundle* or instrument* or care plan* or multi-factorial or multifactorial)) ) 380

S26 TI ( (information n3 (technolog* or system or computeri* or electronic)) ) OR AB ( (information n3 (technolog* or system or computeri* or electronic)) ) 29,460

S25 TI ( (electronic n2 record*) or digital* ) OR AB ( (electronic n2 record*) or digital* ) 66,503

S24 (MH "Clinical Assessment Tools+") 252,835

S23 TI (assess* n4 tool*) OR AB (assess* n4 tool*) 31,239

S22 (MH "Fall Risk Assessment Tool") 164

S21 S16 OR S17 OR S18 OR S19 OR S20 384,391

S20 (MH "Guideline Adherence") 15,898

S19 (MH "Systems Implementation") OR (MH "Program Implementation") OR (MH "Implementation Science") 30,900

S18 TI ( engage* or "buy in" or (Cognitive n2 participat*) ) OR AB ("buy in" or (Cognitive n2 participat*) ) 16,158

S17 TI ( sustain* n4 (program* or practice or practices or intervention or interventions) ) OR AB ( sustain* n4 (program* or practice or practices or intervention or interventions) ) 6,822

S16 TI ( implement* or adopt* or deliver* or adher* or comply or complian* or fidelity ) OR AB ( implement* or adher* or comply or complian* or fidelity) 344,120

S15 S13 OR S14 18,729

S14 TI (fall* n2 (assess* or risk* or prevent* or reduc* or occur* or frequen* or screen*)) OR AB (fall* n2 (assess* or risk* or prevent* or reduc* or occur* or frequen* or screen*)) 14,235

S13 (MH "Accidental Falls/PC") 10,323

S12 S1 OR S2 OR S3 OR S4 OR S5 OR S6 OR S7 OR S8 OR S9 OR S10 OR S11 704,077

S11 (MH "Multidisciplinary Care Team+") 46,811

S10 (MH "Medical Staff, Hospital+") OR (MH "Nursing Staff, Hospital") 27,035

S9 (MH "Hospitals+") 121,072

S8 (MH "Hospital Units") OR (MH "Rehabilitation Centers") 15,793

S7 (MH "Inpatients") 83,955

S6 (MH "Subacute Care") OR (MH "Acute Care") 10,376

S5 (MH "Hospitalization") 38,763

S4 TI hospital* OR AB hospital* 476,803

S3 TI ( inpatient or inpatients) OR AB ( inpatient or inpatients) 54,348

S2 TI ( ((rehabilitation or geriatric) n1 (ward? or unit? or department?)) ) OR AB ( ((rehabilitation or geriatric) n1 (ward? or unit? or department?)) ) 4,834

S1 TI ( ((acute or sub-acute or subacute) n3 (care or ward* or patient*)) ) OR AB ( ((acute or sub-acute or subacute) n3 (care or ward* or patient*)) ) 73,897

**Embase Classic+Embase  (Ovid) 1947 to 2021 May 05**

**Search Date: 06/05/2021**

**Records found: 45**

**Embase Classic+Embase <1947 to 2021 May 05>**

**Search Strategy:**

--------------------------------------------------------------------------------

1     hospitalization/ (420992)

2     hospital department/ or exp ward/ (439715)

3     exp hospital/ (1302934)

4     medical staff/ (40310)

5     nursing staff/ (74427)

6     rehabilitation center/ (17387)

7     subacute care/ (1151)

8     exp clinical handover/ or collaborative care team/ or exp rapid response team/ (11414)

9     exp hospital patient/ (199241)

10     ((acute or sub-acute or subacute) adj3 (care or ward?)).tw,kw. (51102)

11     ((rehabilitation or geriatric) adj (ward? or unit? or department?)).tw,kw. (12324)

12     inpatient?.tw,kw. (199814)

13     (acute adj5 patient?).tw,kw. (369927)

14     hospital*.tw,kw. (2200719)

15     or/1-14 [hospital] (3282770)

16     *falling/ (12578)

17     falling/pc (2954)

18     (fall? adj2 (assess* or risk? or prevent* or reduc* or occur* or frequen* or screen*)).tw,kw. (25940)

19     or/16-18 [Falls prevention or risk assessment] (32853)

20     implement*.tw,kw. (724768)

21     (sustain* adj4 (program* or practice* or intervention?)).tw,kw. (14660)

22     Adopt*.tw,kw. /freq=2 (49567)

23     Adopt*.ti. (20665)

24     deliver*.tw,kw. /freq=2 (374349)

25     (adher* or comply or complian*).tw,kw. (526988)

26     fidelity.tw,kw. (35433)

27     implementation science/ (1982)

28     exp protocol compliance/ (15723)

29     engagement.tw,kw. (97441)

30     engage*.tw,kw. /freq=2 (52149)

31     (intervention adj2 deliver*).tw,kw. (7304)

32     (Cognitive adj2 participat*).tw,kw. (729)

33     "buy in".tw,kw. (2788)

34     or/20-33 [Engagement or Implementation] (1725436)

35     clinical assessment tool/ (25003)

36     (assess* adj4 tool?).tw,kw. (94602)

37     (electronic adj2 record?).tw,kw. (83628)

38     *fall risk assessment/ (605)

39     medical informatics/ or nursing informatics/ (23031)

40     exp hospital information system/ (25135)

41     exp information technology device/ (201010)

42     (information adj3 (technolog* or system or computeri* or electronic)).tw,kw. (70350)

43     digital*.tw,kw. (211640)

44     ("risk assess*" adj4 (bundle* or instrument* or care plan*)).tw,kw. (604)

45     ((multi-factorial or multifactorial) adj4 risk assessment*).tw,kw. (130)

46     (risk* adj3 screen* adj3 tool*).tw,kw. (1181)

47     clinical decision support system/ (3800)

48     clinic* decision* support system*.tw,kw. (3087)

49     health information management.tw,kw. (909)

50     medical information system/ or bedside information system/ (22022)

51     (electronic* adj3 nurs* adj3 document*).tw,kw. (137)

52     or/35-51 [Assessment tools including health info technology] (692085)

53     workflow/ (26854)

54     integration/ (6459)

55     data integration/ (544)

56     facilitation.tw,kw. (39629)

57     (workflow* or work-flow*).tw,kw. (43560)

58     system? fit*.tw,kw. (459)

59     Embed*.tw,kw. (176919)

60     integrat*.tw,kw. (705509)

61     (routine* or routini*).tw,kw. (628385)

62     or/53-61 [Workflows concept] (1543566)

63     15 and 19 and 34 and 52 and 62 [HIT tools CMO4 - final] (45)

**Ovid MEDLINE(R) ALL 1946 to May 05, 2021**

**Search Date: 06/05/2021**

**Records found: 30**

**Ovid MEDLINE(R) ALL <1946 to May 05, 2021>**

**Search Strategy:**

--------------------------------------------------------------------------------

1     Hospitalization/ (115592)

2     Subacute Care/ (1169)

3     Hospital Units/ (10319)

4     exp Hospitals/ (284007)

5     medical staff, hospital/ or nursing staff, hospital/ (66506)

6     exp Patient Care Team/ (70042)

7     Rehabilitation Centers/ (8356)

8     Inpatients/ (23637)

9     ((acute or sub-acute or subacute) adj3 (care or ward?)).tw,kf. (34662)

10     ((rehabilitation or geriatric) adj (ward? or unit? or department?)).tw,kf. (6803)

11     (acute adj5 patient?).tw,kf. (227174)

12     inpatient?.tw,kf. (116838)

13     hospital*.tw,kf. (1383898)

14     or/1-13 [hospital] (1835443)

15     Accidental Falls/pc [Prevention & Control] (9549)

16     (fall? adj2 (assess* or risk? or prevent* or reduc* or occur* or frequen* or screen*)).tw,kf. (17142)

17     15 or 16 [Falls] (21139)

18     implement*.tw,kf. (545160)

19     (sustain* adj4 (program* or practice* or intervention?)).tw,kf. (11220)

20     Adopt*.tw,kf. /freq=2 (37248)

21     Adopt*.ti. (16394)

22     deliver*.tw,kf. /freq=2 (258583)

23     deliver*.ti. (137239)

24     (adher* or comply or complian*).tw,kf. (343916)

25     fidelity.tw,kf. (30445)

26     Health Plan Implementation/ (6379)

27     implementation science/ or technology transfer/ (2784)

28     Guideline Adherence/ (33374)

29     engage*.tw,kf. /freq=2 (39474)

30     engage*.ti. (17196)

31     (intervention adj2 deliver*).tw,kf. (5693)

32     (Cognitive adj2 participat*).tw,kf. (547)

33     "buy in".tw,kf. (1792)

34     or/18-33 [Implementation or Adherence to Guidelines and strategies] (1263268)

35     Risk Assessment/mt [Methods] (34919)

36     (assess* adj4 tool?).tw,kf. (64154)

37     (electronic adj2 record?).tw,kf. (43674)

38     medical informatics/ or health information exchange/ or exp medical informatics applications/ or exp medical informatics computing/ or nursing informatics/ (460638)

39     exp Hospital Information Systems/ (28271)

40     exp health information management/ (1788)

41     exp Medical Records Systems, Computerized/ (42909)

42     (information adj3 (technolog* or system or computeri* or electronic)).tw,kf. (52606)

43     digital*.tw,kf. (157475)

44     ("risk assess*" adj4 (bundle* or instrument* or care plan*)).tw,kf. (487)

45     clinic* decision* support system*.tw,kf. (2415)

46     (electronic* adj3 nurs* adj3 document*).tw,kf. (118)

47     Decision Support Systems, Clinical/ (8422)

48     or/35-47 [Assessment tools including health IT] (787014)

49     workflow/ (6346)

50     systems integration/ (9571)

51     facilitation.tw,kf. (30997)

52     (workflow* or work-flow*).tw,kf. (29615)

53     system? fit*.tw,kf. (333)

54     Embed*.tw,kf. (138578)

55     integrat*.tw,kf. (568134)

56     (routine* or routini*).tw,kf. (405465)

57     or/49-56 [Workflows] (1141149)

58     14 and 17 and 34 and 48 and 57 [Facilitation Tools CMO 4] (30)

**NICE Evidence**

**Search Date: 07/05/2021**

Records found: 43

("falls prevention" or "falls assessment" or "falls reduction") and (hospital or inpatient or "acute care" or "acute ward" or "acute patient" or "rehabilitation unit" or "rehabilitation ward" ) and ("clinical decision support system" or "assessment tool") and (workflow or routine or "system fit")

Sorted by Relevance .| Sort by Date

Filters applied:

Evidence type : Evidence Summaries

Evidence type: Primary Research

Evidence type: Systematic Reviews

Evidence type: Audit and Inspection Reports

Evidence type: Health Technology Assessments

**Web of Science Core Collection**

**Databases available at the University of Leeds (searched simultaneously):  Arts & Humanities Citation Index (Web of Science) 1975-present**

- Conference Proceedings Citation Index- Science ( Web of Science) 1990-present
- Conference Proceedings Citation Index- Social Science & Humanities (Web of Science) 1990-present
- Science Citation Index-Expanded (Web of Science) 1900-present
- Social Sciences Citation Index (Web of Science) 1900-present
- Emerging Sources Citation Index (Web of Science) 2015-present

**Search Date: 06/05/2021**

Records found: 25

Indexes=SCI-EXPANDED, SSCI, A&HCI, CPCI-S, CPCI-SSH, ESCI Timespan=1900-2021

# 24 25 #23 AND #22 AND #10 AND #6 AND #5

# 23 2,857,583 TOPIC: (facilitation or workflow* or work-flow* or embed* or integrat* or routine* or routini* or "system* fit*")

# 22 1,106,013 #21 OR #20 OR #19 OR #18 OR #17 OR #16 OR #15 OR #14 OR #13 OR #12 OR #11

# 21 151 TOPIC: (electronic* near/3 nurs* near/3 document*)

# 20 606 TOPIC: ("health information management")

# 19 3,188 TOPIC: ("clinic* decision* support system*")

# 18 1,302 TOPIC: (risk* near/3 screen* near/3 tool*)

# 17 1,074 TOPIC: ((multi-factorial or multifactorial) near/4 risk assessment*)

# 16 21 TS=("risk assess*" near/4 ("care plan*") )

# 15 739 TS=("risk assess*" near/4 (bundle* or instrument*) )

# 14 656,213 TOPIC: (digital*)

# 13 46,322 TOPIC: (electronic near/2 record$)

# 12 337,953 TOPIC: (information near/3 (technolog* or system or computeri* or electronic) )

# 11 99,734 TOPIC: (assess* near/4 tool$)

# 10 2,791,484 #9 OR #8 OR #7

# 9 30,828 TOPIC: (sustain* near/4 (program* or practice* or intervention$) )

# 8 296,145 TI=(adopt* or deliver* or engage*)

# 7 2,496,599 TS=(implement* or adher* or comply or complian* or fidelity or "buy in" or (Cognitive near/2 participat*) )

# 6 28,263 TS=(fall$ near/2 (assess* or risk$ or prevent* or reduc* or occur* or frequen* or screen*) )

# 5 1,439,906 #4 OR #3 OR #2 OR #1

# 4 225,427 TOPIC: (acute near/5 patient$)

# 3 7,521 TOPIC: ((rehabilitation or geriatric) near/1 (ward$ or unit$ or department$) )

# 2 35,952 TOPIC: ((acute or sub-acute or subacute) near/3 (care or ward$) )

# 1 1,254,650 TOPIC: (hospital* OR inpatient*)

***Shared responsibility/teamwork***

**CINAHL (EBSCOhost)**

**Search Date: 06/05/2021**

**Records found: 42**

S36 S12 AND S15 AND S20 AND S28 AND S35 42

S35 S29 OR S30 OR S31 OR S32 OR S33 OR S34   439,642

S34 TI ( (responsib* n3 (staff or professional or professionals or nurs* or doctor* or clinician*)) ) OR AB ( (responsib* n3 (staff or professional or professionals or nurs* or doctor* or clinician*)) ) 8,563

S33 (MH "Social Responsibility+")   30,941

S32 (MH "Interpersonal Relations+")   293,747

S31 TI ( (ownership or communicat* or vigilan*) ) OR AB ( (ownership or communicat* or vigilan*) ) 143,611

S30 TI ( (joint* n3 (responsibility or responsible or accountability or accountable)) ) OR AB ( (joint* n3 (responsibility or responsible or accountability or accountable)) ) 227

S29 TI ( ((share* or sharing) n3 (responsibility or responsible or accountability or accountable)) ) OR AB ( ((share* or sharing) n3 (responsibility or responsible or accountability or accountable)) ) 1,788

S28 S21 OR S22 OR S23 OR S24 OR S25 OR S26 OR S27   134,358

S27 TI ( (team* n5 (huddle* or handover*)) or teamwork* or "team work*" ) OR AB ( (team* n5 (huddle* or handover*)) or teamwork* or "team work*" ) 9,804

S26 (MH "Interdepartmental Relations") OR (MH "Public Relations") 5,253

S25 (MH "Teamwork")   16,113

S24 TI ( (team* n3 (share* or sharing or communic* or multidisciplin* or interdisciplin* or multi-disciplin* or inter-disciplin* or interprofessional* or inter-professional*)) ) OR AB ( (team* n3 (share* or sharing or communic* or multidisciplin* or interdisciplin* or multi-disciplin* or inter-disciplin* or interprofessional* or inter-professional*)) )   23,302

S23 TI collaborat*   22,997

S22 (MH "Collaboration")   46,618

S21 (MH "Multidisciplinary Care Team+")   46,806

S20 S16 OR S17 OR S18 OR S19   245,854

S19 TI ( (risk n2 (assess* or evaluat*)) or guideline* or protocol* ) AND AB ( (risk n2 (assess* or evaluat*)) or guideline* or protocol* )   33,722

S18 (MH "Protocols+")   42,093

S17 (MH "Practice Guidelines")   81,196

S16 (MH "Risk Assessment")    114,426

S15 S13 OR S14   18,732

S14 TI (fall* n2 (assess* or risk* or prevent* or reduc* or occur* or frequen* or screen*)) OR AB (fall* n2 (assess* or risk* or prevent* or reduc* or occur* or frequen* or screen*))   14,238

S13 (MH "Accidental Falls/PC")   10,324

S12 S1 OR S2 OR S3 OR S4 OR S5 OR S6 OR S7 OR S8 OR S9 OR S10 OR S11   703,827

S11 (MH "Multidisciplinary Care Team+")   46,806

S10 (MH "Medical Staff, Hospital+") OR (MH "Nursing Staff, Hospital")   27,034

S9 (MH "Hospitals+")   121,025

S8 (MH "Hospital Units") OR (MH "Rehabilitation Centers")   15,791

S7 (MH "Inpatients")   83,940

S6 (MH "Subacute Care") OR (MH "Acute Care")   10,379

S5 (MH "Hospitalization")   38,752

S4 TI hospital* OR AB hospital*   476,571

S3 TI ( inpatient or inpatients) OR AB ( inpatient or inpatients)   54,338

S2 TI ( ((rehabilitation or geriatric) n1 (ward? or unit? or department?)) ) OR AB ( ((rehabilitation or geriatric) n1 (ward? or unit? or department?)) ) 4,830

S1 TI ( ((acute or sub-acute or subacute) n3 (care or ward* or patient*)) ) OR AB ( ((acute or sub-acute or subacute) n3 (care or ward* or patient*)) )   73,873

**Embase Classic+Embase  (Ovid) 1947 to 2021 May 05**

**Search Date: 06/05/2021**

**Records found: 42**

**Embase Classic+Embase <1947 to 2021 May 05>**

**Search Strategy:**

--------------------------------------------------------------------------------

1     hospitalization/ (420992)

2     hospital department/ or exp ward/ (439715)

3     exp hospital/ (1302934)

4     medical staff/ (40310)

5     nursing staff/ (74427)

6     rehabilitation center/ (17387)

7     subacute care/ (1151)

8     exp clinical handover/ or collaborative care team/ or exp rapid response team/ (11414)

9     exp hospital patient/ (199241)

10     ((acute or sub-acute or subacute) adj3 (care or ward?)).tw,kw. (51102)

11     ((rehabilitation or geriatric) adj (ward? or unit? or department?)).tw,kw. (12324)

12     inpatient?.tw,kw. (199814)

13     (acute adj5 patient?).tw,kw. (369927)

14     hospital*.tw,kw. (2200719)

15     or/1-14 [hospital] (3282770)

16     *falling/ (12578)

17     falling/pc (2954)

18     (fall? adj2 (assess* or risk? or prevent* or reduc* or occur* or frequen* or screen*)).tw,kw. (25940)

19     or/16-18 [Falls prevention or risk assessment] (32853)

20     risk assessment/ (618392)

21     (risk adj2 (assess* or evaluat*)).tw,kw. (196115)

22     exp practice guideline/ (601575)

23     guideline*.tw,kw. (613857)

24     exp clinical protocol/ (106751)

25     protocol?.tw,kw. /freq=2 (185559)

26     or/20-25 [Multifactorial risk assessment] (1743357)

27     multidisciplinary team/ or collaborative care team/ (12645)

28     collaborat*.tw,kw. /freq=2 (50596)

29     collaborat*.ti. (40601)

30     (team* adj3 (share* or sharing or communic*)).tw,kw. (7724)

31     (team* adj3 (multidisciplin* or interdisciplin* or multi-disciplin* or inter-disciplin* or interprofessional* or inter-professional*)).tw,kw. (61882)

32     cooperation/ or teamwork/ (64251)

33     public relations/ (62742)

34     (team* adj5 huddle?).tw,kw. (337)

35     (team* adj5 handover?).tw,kw. (230)

36     (teamwork* or team-work*).tw,kw. (20866)

37     or/27-36 [Teamwork] (247513)

38     ((share* or sharing) adj3 (responsibility or responsible or accountability or accountable)).tw,kw. (3181)

39     (joint adj3 (responsibility or responsible or accountability or accountable)).tw,kw. (586)

40     ownership.tw,kw. (17297)

41     communicat*.tw,kw. (442253)

42     exp interpersonal communication/ (717292)

43     *"organization and management"/ (65602)

44     alertness/ (19538)

45     vigilan*.tw,kw. (32155)

46     (responsib* adj3 (staff or professional? or nurs* or doctor? or clinician?)).tw,kw. (11898)

47     or/38-46 [Shared Responsibility] (1148502)

48     15 and 19 and 26 and 37 and 47 [Falls in Hospital and Risk Assessment and Teamwork and Shared Responsibility] (42)

**Ovid MEDLINE(R) ALL 1946 to May 05, 2021**

**Search Date: 06/05/2021**

**Records found: 55**

 Search Strategy:

--------------------------------------------------------------------------------

1     Hospitalization/ (115592)

2     Subacute Care/ (1169)

3     Hospital Units/ (10319)

4     exp Hospitals/ (284007)

5     medical staff, hospital/ or nursing staff, hospital/ (66506)

6     exp Patient Care Team/ (70042)

7     Rehabilitation Centers/ (8356)

8     Inpatients/ (23637)

9     ((acute or sub-acute or subacute) adj3 (care or ward?)).tw,kf. (34662)

10     ((rehabilitation or geriatric) adj (ward? or unit? or department?)).tw,kf. (6803)

11     inpatient?.tw,kf. (116838)

12     (acute adj5 patient?).tw,kf. (227174)

13     hospital*.tw,kf. (1383898)

14     or/1-13 [hospital] (1835443)

15     Accidental Falls/pc [Prevention & Control] (9549)

16     (fall? adj2 (assess* or risk? or prevent* or reduc* or occur* or frequen* or screen*)).tw,kf. (17142)

17     15 or 16 [Falls] (21139)

18     exp Risk Assessment/ (284216)

19     (risk adj2 (assess* or evaluat*)).tw,kf. (137873)

20     guideline/ or practice guideline/ (35660)

21     guideline*.tw,kf. (386469)

22     protocol?.tw,kf. /freq=2 (126031)

23     protocol?.ti. (69596)

24     exp Clinical Protocols/ (174448)

25     or/18-24 [Multifactorial risk assessment] (1055597)

26     exp Patient Care Team/ (70042)

27     Cooperative Behavior/ (44475)

28     collaborat*.tw,kf. /freq=2 (35346)

29     collaborat*.ti. (32563)

30     (team* adj3 (share* or sharing or communic*)).tw,kf. (4639)

31     (team* adj3 (multidisciplin* or interdisciplin* or multi-disciplin* or inter-disciplin* or interprofessional* or inter-professional*)).tw,kf. (34335)

32     exp interprofessional relations/ or physician-nurse relations/ (70564)

33     (team* adj5 huddle?).tw,kf. (106)

34     (team* adj5 handover?).tw,kf. (115)

35     (teamwork* or team-work*).tw,kf. (14050)

36     or/26-35 [Teamwork] (228807)

37     ((share* or sharing) adj3 (responsibility or responsible or accountability or accountable)).tw,kf. (2440)

38     (joint* adj3 (responsibility or responsible or accountability or accountable)).tw,kf. (604)

39     ownership.tw,kf. (13717)

40     communicat*.tw,kf. (325944)

41     exp Interpersonal Relations/ (334498)

42     exp *"Organization and Administration"/ (729180)

43     vigilan*.tw,kf. (22242)

44     (responsib* adj3 (staff or professional? or nurs* or doctor? or clinician?)).tw,kf. (8985)

45     or/37-44 [Shared Responsibility] (1323953)

46     14 and 17 and 25 and 36 and 45 [Falls in Hospital and Risk Assessment and Teamwork and Shared Responsibility] (55)

**NICE Evidence**

**Search Date: 07/05/2021**

**Records found: 27**

("falls prevention" or "falls assessment" or "falls reduction") and (hospital or inpatient or "acute care" or "acute ward" or "acute patient" or "rehabilitation unit" or "rehabilitation ward" ) and ("team huddle" or "team handover" or "interdisciplinary team" or "team share" or teamwork or interprofessional or "care team") and (communication or collaboration or responsibility or ownership or accountability) and ("risk assessment" or "risk evaluation" or "evaluation of risk")

Sorted by Relevance .| Sort by Date

Filters applied:

Evidence type : Evidence Summaries

Evidence type: Primary Research

Evidence type: Systematic Reviews

Evidence type: Audit and Inspection Reports

Evidence type: Health Technology Assessments

**Web of Science Core Collection**

**Databases available at the University of Leeds (searched simultaneously):**

- Arts & Humanities Citation Index (Web of Science) 1975-present
- Conference Proceedings Citation Index- Science ( Web of Science) 1990-present
- Conference Proceedings Citation Index- Social Science & Humanities (Web of Science) 1990-present
- Science Citation Index-Expanded (Web of Science) 1900-present
- Social Sciences Citation Index (Web of Science) 1900-present
- Emerging Sources Citation Index (Web of Science) 2015-present

**Search Date: 06/05/2021**

Records found: 9

Indexes=SCI-EXPANDED, SSCI, A&HCI, CPCI-S, CPCI-SSH, ESCI Timespan=1900-2021

# 20 9 #19 AND #14 AND #9 AND #6 AND #5

# 19 1,273,025 #18 OR #17 OR #16 OR #15

# 18 9,478 TOPIC: ((responsib* near/3 (staff or professional$ or nurs* or doctor$ or clinician$) ))

# 17 1,258,633 TOPIC: (ownership or communicat* or vigilan*)

# 16 1,068 TS=(joint* near/3 (responsibility or responsible or accountability or accountable) )

# 15 6,067 TOPIC: (((share* or sharing) near/3 (responsibility or responsible or accountability or accountable) ))

# 14 455,274 #13 OR #12 OR #11 OR #10

# 13 228 TOPIC: ((team* near/5 (huddle$ or handover$) ))

# 12 34,696 TOPIC: ((team* near/3 (multidisciplin* or interdisciplin* or multi-disciplin* or inter-disciplin* or interprofessional* or inter-professional*) ))

# 11 10,763 TOPIC: ((team* near/3 (share* or sharing or communic*) ))

# 10 422,965 TS=(collaborat* or teamwork* or "team work*")

# 9 1,540,975 #8 OR #7

# 8 1,298,433 TOPIC: (guideline* or protocol*)

# 7 267,127 TOPIC: (risk near/2 (assess* or evaluat*) )

# 6 28,265 TS=(fall$ near/2 (assess* or risk$ or prevent* or reduc* or occur* or frequen* or screen*) )

# 5 1,439,877 #4 OR #3 OR #2 OR #1

# 4 225,435 TOPIC: (acute near/5 patient$)

# 3 7,521 TOPIC: ((rehabilitation or geriatric) near/1 (ward$ or unit$ or department$) )

# 2 35,954 TOPIC: ((acute or sub-acute or subacute) near/3 (care or ward$) )

# 1 1,254,611 TOPIC: (hospital* OR inpatient*)

***Patient Participation***

**CINAHL (EBSCOhost)**

**Search Date: 06/05/2021**

**Records found: 35**

# Query Results

S37 S12 AND S15 AND S20 AND S29 AND S36 35

S36 S30 OR S31 OR S32 OR S33 OR S34 OR S35 829,085

S35 TI ( (compassion* or listen* or respect* or empower* or communicat*) ) OR AB ( (compassion* or listen* or respect* or empower* or communicat*) ) 569,095

S34 (MH "Empowerment") 14,502

S33 (MH "Empathy") OR (MH "Respect") 14,446

S32 (MH "Interpersonal Relations+") 293,759

S31 (MH "Listening") 3,900

S30 (MH "Compassion") 2,156

S29 S21 OR S22 OR S23 OR S24 OR S25 OR S26 OR S27 OR S28 189,301

S28 (MH "Patient Autonomy") 6,773

S27 TI ( ((person-centred or person-centered or individuali* or personali*) n2 care) ) OR AB ( ((person-centred or person-centered or individuali* or personali*) n2 care) ) 7,646

S26 TI ( (multifacet* or multi-facet* or tailor*) ) OR AB ( (multifacet* or multi-facet* or tailor*) ) 39,589

S25 TI ( (engag* n3 (patient? or famil* or carer? or caregiver? or player*)) ) OR AB ( (engag* n3 (patient? or famil* or carer? or caregiver? or player*)) ) 9,745

S24 (MH "Consumer Participation") 21,098

S23 TI ( (patient or patients) n5 (need* or perspective* or autonom*) ) OR AB ( (patient or patients) n5 (need* or perspective* or autonom*) ) 81,650

S22 TI ( ((patient or patients) n2 (centred or centered)) ) OR AB ( ((patient or patients) n2 (centred or centered)) ) 16,711

S21 (MH "Patient Centered Care") OR (MH "Shared Medical Appointments") 31,822

S20 S16 OR S17 OR S18 OR S19 245,878

S19 TI ( (risk n2 (assess* or evaluat*)) or guideline* or protocol* ) AND AB ( (risk n2 (assess* or evaluat*)) or guideline* or protocol* ) 33,732

S18 (MH "Protocols+") 42,099

S17 (MH "Practice Guidelines") 81,201

S16 (MH "Risk Assessment") 114,437

S15 S13 OR S14 18,729

S14 TI (fall* n2 (assess* or risk* or prevent* or reduc* or occur* or frequen* or screen*)) OR AB (fall* n2 (assess* or risk* or prevent* or reduc* or occur* or frequen* or screen*)) 14,235

S13 (MH "Accidental Falls/PC") 10,323

S12 S1 OR S2 OR S3 OR S4 OR S5 OR S6 OR S7 OR S8 OR S9 OR S10 OR S11 704,077

S11 (MH "Multidisciplinary Care Team+") 46,811

S10 (MH "Medical Staff, Hospital+") OR (MH "Nursing Staff, Hospital") 27,035

S9 (MH "Hospitals+") 121,072

S8 (MH "Hospital Units") OR (MH "Rehabilitation Centers") 15,793

S7 (MH "Inpatients") 83,955

S6 (MH "Subacute Care") OR (MH "Acute Care") 10,376

S5 (MH "Hospitalization") 38,763

S4 TI hospital* OR AB hospital* 476,803

S3 TI ( inpatient or inpatients) OR AB ( inpatient or inpatients) 54,348

S2 TI ( ((rehabilitation or geriatric) n1 (ward? or unit? or department?)) ) OR AB ( ((rehabilitation or geriatric) n1 (ward? or unit? or department?)) ) 4,834

S1 TI ( ((acute or sub-acute or subacute) n3 (care or ward* or patient*)) ) OR AB ( ((acute or sub-acute or subacute) n3 (care or ward* or patient*)) )

**Embase Classic+Embase  (Ovid) 1947 to 2021 May 05**

**Search Date: 06/05/2021**

**Records found: 47**

Search Strategy:

--------------------------------------------------------------------------------

1     hospitalization/ (420992)

2     hospital department/ or exp ward/ (439715)

3     exp hospital/ (1302934)

4     medical staff/ (40310)

5     nursing staff/ (74427)

6     rehabilitation center/ (17387)

7     subacute care/ (1151)

8     exp clinical handover/ or collaborative care team/ or exp rapid response team/ (11414)

9     exp hospital patient/ (199241)

10     ((acute or sub-acute or subacute) adj3 (care or ward?)).tw,kw. (51102)

11     ((rehabilitation or geriatric) adj (ward? or unit? or department?)).tw,kw. (12324)

12     inpatient?.tw,kw. (199814)

13     (acute adj5 patient?).tw,kw. (369927)

14     hospital*.tw,kw. (2200719)

15     or/1-14 [hospital] (3282770)

16     *falling/ (12578)

17     falling/pc (2954)

18     (fall? adj2 (assess* or risk? or prevent* or reduc* or occur* or frequen* or screen*)).tw,kw. (25940)

19     or/16-18 [Falls prevention or risk assessment] (32853)

20     risk assessment/ (618392)

21     (risk adj2 (assess* or evaluat*)).tw,kw. (196115)

22     exp practice guideline/ (601575)

23     guideline*.tw,kw. (613857)

24     exp clinical protocol/ (106751)

25     protocol?.tw,kw. /freq=2 (185559)

26     or/20-25 [Multifactorial risk assessment] (1743357)

27     patient care/ (317371)

28     shared medical appointment/ (137)

29     (patient? adj2 (centred or centered)).tw,kw. (36785)

30     (patient? adj5 perspective?).tw,kw. (33299)

31     (patient? adj4 need?).tw,kw. (123089)

32     patient participation/ (29364)

33     (engag* adj3 (patient? or famil* or carer? or caregiver? or player*)).tw,kw. (20011)

34     (multifacet* or multi-facet* or tailor*).tw,kw. (164230)

35     ((person-centred or person-centered) adj2 care).tw,kw. (3453)

36     patient autonomy/ (6173)

37     (patient? adj3 autonom*).tw,kw. (12059)

38     ((individuali* or personali*) adj2 care).tw,kw. (8927)

39     or/27-38 [New Patient Centred Care] (689386)

40     empathy/ (28635)

41     compassion*.tw,kw. (16029)

42     exp interpersonal communication/ (717292)

43     listen*.tw,kw. (42836)

44     respect/ (1344)

45     (empower* adj3 (staff or professional? or nurs* or doctor? or clinician? or patient?)).tw,kw. (9706)

46     empowerment/ (10858)

47     respect*.tw,kw. /freq=2 (1001127)

48     respect*.ti. (14093)

49     communicat*.tw,kw. /freq=2 (122590)

50     communicat*.ti. (83765)

51     or/40-50 [Compassion Empowerment] (1877721)

52     15 and 19 and 26 and 39 and 51 [Falls in Hospital and Risk Assessment and Person-centred care and Empowerment Compassion] (47)

**Ovid MEDLINE(R) ALL 1946 to May 05, 2021**

**Search Date: 06/05/2021**

**Records found: 20**

Search Strategy:

--------------------------------------------------------------------------------

1     Hospitalization/ (115592)

2     Subacute Care/ (1169)

3     Hospital Units/ (10319)

4     exp Hospitals/ (284007)

5     medical staff, hospital/ or nursing staff, hospital/ (66506)

6     exp Patient Care Team/ (70042)

7     Rehabilitation Centers/ (8356)

8     Inpatients/ (23637)

9     ((acute or sub-acute or subacute) adj3 (care or ward?)).tw,kf. (34662)

10     ((rehabilitation or geriatric) adj (ward? or unit? or department?)).tw,kf. (6803)

11     (acute adj5 patient?).tw,kf. (227174)

12     inpatient?.tw,kf. (116838)

13     hospital*.tw,kf. (1383898)

14     or/1-13 [hospital] (1835443)

15     Accidental Falls/pc [Prevention & Control] (9549)

16     (fall? adj2 (assess* or risk? or prevent* or reduc* or occur* or frequen* or screen*)).tw,kf. (17142)

17     15 or 16 [Falls] (21139)

18     exp Risk Assessment/ (284216)

19     (risk adj2 (assess* or evaluat*)).tw,kf. (137873)

20     guideline/ or practice guideline/ (35660)

21     guideline*.tw,kf. (386469)

22     protocol?.tw,kf. /freq=2 (126031)

23     protocol?.ti. (69596)

24     exp Clinical Protocols/ (174448)

25     or/18-24 [Multifactorial risk assessment] (1055597)

26     exp Patient-Centered Care/ (21417)

27     Shared Medical Appointments/ (33)

28     (patient? adj2 (centred or centered)).tw,kf. (26112)

29     (patient? adj5 perspective?).tw,kf. (22183)

30     (patient? adj4 need?).tw,kf. (73644)

31     Patient Participation/ (26982)

32     (engag* adj3 (patient? or famil* or carer? or caregiver? or player*)).tw,kf. (13206)

33     (multifacet* or multi-facet* or tailor*).tw,kf. (128445)

34     (person adj2 (centred or centered)).tw,kf. (6622)

35     personal autonomy/ (17428)

36     autonom*.tw,kf. (147232)

37     ((individuali* or personali*) adj2 care).tw,kf. (6083)

38     or/26-37 [Patient Centred Care] (446228)

39     Empathy/ (19888)

40     compassion*.tw,kf. (11571)

41     listen*.tw,kf. (35198)

42     exp Interpersonal Relations/ (334498)

43     respect*.tw,kf. /freq=2 (655328)

44     respect*.ti. (11346)

45     respect/ (443)

46     empower*.tw,kf. (29195)

47     empowerment/ (409)

48     communicat*.tw,kf. /freq=2 (90369)

49     communicat*.ti. (68439)

50     or/39-49 [Compassion Empowerment] (1165982)

51     14 and 17 and 25 and 38 and 50 [Falls in Hospital and Risk Assessment and Person-centred care and Empowerment Compassion] (20)

**NICE Evidence**

**Search Date: 07/05/2021**

**Records found: 12**

("falls prevention" or "falls assessment" or "falls reduction") and (hospital or inpatient or "acute care" or "acute ward" or "acute patient" or "rehabilitation unit" or "rehabilitation ward" ) and ("patient centered" or "patient centered" or patient centred or patient centered multifaceted or tailored or autonomy or "patient participation") and ("risk assessment" or "risk evaluation" or "evaluation of risk")

Sorted by Relevance .| Sort by Date

Filters applied:

Evidence type : Evidence Summaries

Evidence type: Primary Research

Evidence type: Systematic Reviews

Evidence type: Audit and Inspection Reports

Evidence type: Health Technology Assessments

**Web of Science Core Collection**

Databases available at the University of Leeds (searched simultaneously):

- Arts & Humanities Citation Index (Web of Science) 1975-present
- Conference Proceedings Citation Index- Science ( Web of Science) 1990-present
- Conference Proceedings Citation Index- Social Science & Humanities (Web of Science) 1990-present
- Science Citation Index-Expanded (Web of Science) 1900-present
- Social Sciences Citation Index (Web of Science) 1900-present
- Emerging Sources Citation Index (Web of Science) 2015-present

**Search Date: 06/05/2021**

Records found: 29

Indexes=SCI-EXPANDED, SSCI, A&HCI, CPCI-S, CPCI-SSH, ESCI Timespan=1900-2021

# 19 29 #18 AND #17 AND #9 AND #6 AND #5

# 18 5,738,974 TOPIC: (compassion* or listen* or respect* or empower* or communicat*)

# 17 398,635 #16 OR #15 OR #14 OR #13 OR #12 OR #11 OR #10

# 16 9,364 TOPIC: (patient$ near/3 autonom*)

# 15 10,186 TS=((person-centred or person-centered or individuali* or personali*) near/2 care)

# 14 227,277 TOPIC: (multifacet* or multi-facet* or tailor*)

# 13 16,446 TOPIC: (engag* near/3 (patient$ or famil* or carer$ or caregiver$ or player*) )

# 12 73,290 TOPIC: (patient$ near/4 need$)

# 11 24,325 TOPIC: (patient$ near/5 perspective$)

# 10 55,014 TOPIC: (patient$ near/2 (centred or centered) )

# 9 1,541,009 #8 OR #7

# 8 1,298,454 TOPIC: (guideline* or protocol*)

# 7 267,143 TOPIC: (risk near/2 (assess* or evaluat*) )

# 6 27,330 TOPIC: (fall$ near/2 (assess* or risk$ or prevent* or reduc* or occur* or frequenc* or screen) )

# 5 1,439,993 #4 OR #3 OR #2 OR #1

# 4 225,441 TOPIC: (acute near/5 patient$)

# 3 7,521 TOPIC: ((rehabilitation or geriatric) near/1 (ward$ or unit$ or department$) )

# 2 35,955 TOPIC: ((acute or sub-acute or subacute) near/3 (care or ward$) )

# 1 1,254,725 TOPIC: (hospital* OR inpatient*)

 Search 2.3: Update search of 2 prioritised theories

***Facilitation***

**CINAHL (EBSCOhost)**

**Search Date: 01/08/2022**

**Records found: 43**

**# Query Results**

S39 S12 AND S15 AND S21 AND S34 AND S38 43

S38 S35 OR S36 OR S37 295,429

S37 TI ( (facilitation or workflow* or work-flow* or embed* or integrat* or routine* or routini* or "system* fit*") ) OR AB ( (facilitation or workflow* or work-flow* or embed* or integrat* or routine* or routini* or "system* fit*") ) 286,828

S36 (MH "Health Care Delivery, Integrated") 13,765

S35 (MH "Systems Integration") OR (MH "Workflow") 5,346

S34 S22 OR S23 OR S24 OR S25 OR S26 OR S27 OR S28 OR S29 OR S30 OR S31 OR S32 OR S33 469,656

S33 (MH "Information Technology+") 20,764

S32 (MH "Medical Informatics") OR (MH "Nursing Informatics") OR (MH "Health Informatics") 12,898

S31 (MH "Clinical Information Systems+") OR (MH "Health Information Systems+") OR (MH "Hospital Information Systems") OR (MH "Nursing Information Systems+") OR (MH "Patient Record Systems+") OR (MH "Health Information Networks") OR (MH "Decision Support Systems, Clinical") 62,889

S30 TI "clinic* decision* support system*" OR AB "clinic* decision* support system*" 1,303

S29 TI (electronic* n3 nurs* n3 document*) OR AB (electronic* n3 nurs* n3 document*) 197

S28 TI (risk* n3 screen* n3 tool*) OR AB (risk* n3 screen* n3 tool*) 781

S27 TI ( ("risk assess*" n4 (bundle* or instrument* or care plan* or multi-factorial or multifactorial)) ) OR AB ( ("risk assess*" n4 (bundle* or instrument* or care plan* or multi-factorial or multifactorial)) ) 412

S26 TI ( (information n3 (technolog* or system or computeri* or electronic)) ) OR AB ( (information n3 (technolog* or system or computeri* or electronic)) ) 33,802

S25 TI ( (electronic n2 record*) or digital* ) OR AB ( (electronic n2 record*) or digital* ) 79,211

S24 (MH "Clinical Assessment Tools+") 276,801

S23 TI (assess* n4 tool*) OR AB (assess* n4 tool*) 36,074

S22 (MH "Fall Risk Assessment Tool") 179

S21 S16 OR S17 OR S18 OR S19 OR S20 433,844

S20 (MH "Guideline Adherence") 16,876

S19 (MH "Systems Implementation") OR (MH "Program Implementation") OR (MH "Implementation Science") 34,426

S18 TI ( engage* or "buy in" or (Cognitive n2 participat*) ) OR AB ("buy in" or (Cognitive n2 participat*) ) 18,593

S17 TI ( sustain* n4 (program* or practice or practices or intervention or interventions) ) OR AB ( sustain* n4 (program* or practice or practices or intervention or interventions) ) 7,956

S16 TI ( implement* or adopt* or deliver* or adher* or comply or complian* or fidelity ) OR AB ( implement* or adher* or comply or complian* or fidelity) 390,241

S15 S13 OR S14 20,543

S14 TI (fall* n2 (assess* or risk* or prevent* or reduc* or occur* or frequen* or screen*)) OR AB (fall* n2 (assess* or risk* or prevent* or reduc* or occur* or frequen* or screen*)) 15,922

S13 (MH "Accidental Falls/PC") 10,958

S12 S1 OR S2 OR S3 OR S4 OR S5 OR S6 OR S7 OR S8 OR S9 OR S10 OR S11 772,650

S11 (MH "Multidisciplinary Care Team+") 49,799

S10 (MH "Medical Staff, Hospital+") OR (MH "Nursing Staff, Hospital") 29,619

S9 (MH "Hospitals+") 128,590

S8 (MH "Hospital Units") OR (MH "Rehabilitation Centers") 16,781

S7 (MH "Inpatients") 85,946

S6 (MH "Subacute Care") OR (MH "Acute Care") 11,259

S5 (MH "Hospitalization") 43,860

S4 TI hospital* OR AB hospital* 532,701

S3 TI ( inpatient or inpatients) OR AB ( inpatient or inpatients) 61,118

S2 TI ( ((rehabilitation or geriatric) n1 (ward? or unit? or department?)) ) OR AB ( ((rehabilitation or geriatric) n1 (ward? or unit? or department?)) ) 5,417

S1 TI ( ((acute or sub-acute or subacute) n3 (care or ward* or patient*)) ) OR AB ( ((acute or sub-acute or subacute) n3 (care or ward* or patient*)) ) 81,585

**Embase Classic+Embase <1947 to 2022 July 29>**

**Search Date: 01/08/2022**

**Records found: 57**

Search Strategy:

--------------------------------------------------------------------------------

1 hospitalization/ (478011)

2 hospital department/ or exp ward/ (500590)

3 exp hospital/ (1406920)

4 medical staff/ (42122)

5 nursing staff/ (76712)

6 rehabilitation center/ (18901)

7 subacute care/ (1508)

8 exp clinical handover/ or collaborative care team/ or exp rapid response team/ (13877)

9 exp hospital patient/ (222261)

10 ((acute or sub-acute or subacute) adj3 (care or ward?)).tw,kw. (54304)

11 ((rehabilitation or geriatric) adj (ward? or unit? or department?)).tw,kw. (13043)

12 inpatient?.tw,kw. (220506)

13 (acute adj5 patient?).tw,kw. (397349)

14 hospital*.tw,kw. (2403565)

15 or/1-14 [hospital] (3569217)

16 *falling/ (13345)

17 falling/pc (2951)

18 (fall? adj2 (assess* or risk? or prevent* or reduc* or occur* or frequen* or screen*)).tw,kw. (28030)

19 or/16-18 [Falls prevention or risk assessment] (35283)

20 implement*.tw,kw. (817689)

21 (sustain* adj4 (program* or practice* or intervention?)).tw,kw. (16587)

22 Adopt*.tw,kw. /freq=2 (53825)

23 Adopt*.ti. (22299)

24 deliver*.tw,kw. /freq=2 (391321)

25 (adher* or comply or complian*).tw,kw. (565478)

26 fidelity.tw,kw. (39034)

27 implementation science/ (2954)

28 exp protocol compliance/ (18558)

29 engagement.tw,kw. (112073)

30 engage*.tw,kw. /freq=2 (59254)

31 (intervention adj2 deliver*).tw,kw. (8426)

32 (Cognitive adj2 participat*).tw,kw. (805)

33 "buy in".tw,kw. (3176)

34 or/20-33 [Engagement or Implementation] (1883705)

35 clinical assessment tool/ (26509)

36 (assess* adj4 tool?).tw,kw. (106630)

37 (electronic adj2 record?).tw,kw. (98321)

38 *fall risk assessment/ (660)

39 medical informatics/ or nursing informatics/ (23891)

40 exp hospital information system/ (27015)

41 exp information technology device/ (214637)

42 (information adj3 (technolog* or system or computeri* or electronic)).tw,kw. (73764)

43 digital*.tw,kw. (236637)

44 ("risk assess*" adj4 (bundle* or instrument* or care plan*)).tw,kw. (663)

45 ((multi-factorial or multifactorial) adj4 risk assessment*).tw,kw. (148)

46 (risk* adj3 screen* adj3 tool*).tw,kw. (1353)

47 clinical decision support system/ (4833)

48 clinic* decision* support system*.tw,kw. (3630)

49 health information management.tw,kw. (998)

50 medical information system/ or bedside information system/ (22768)

51 (electronic* adj3 nurs* adj3 document*).tw,kw. (155)

52 or/35-51 [Assessment tools including health info technology] (763497)

53 workflow/ (33077)

54 integration/ (6797)

55 data integration/ (1220)

56 facilitation.tw,kw. (40639)

57 (workflow* or work-flow*).tw,kw. (53221)

58 system? fit*.tw,kw. (493)

59 Embed*.tw,kw. (191639)

60 integrat*.tw,kw. (778087)

61 (routine* or routini*).tw,kw. (673222)

62 or/53-61 [Workflows concept] (1679788)

63 15 and 19 and 34 and 52 and 62 [HIT tools CMO4 - final] (57) Medline

**Ovid MEDLINE(R) ALL <1946 to July 29, 2022>**

**Search Date: 01/08/2022**

**Records found: 38**

Search Strategy:

--------------------------------------------------------------------------------

1 Hospitalization/ (129166)

2 Subacute Care/ (1351)

3 Hospital Units/ (10446)

4 exp Hospitals/ (306482)

5 medical staff, hospital/ or nursing staff, hospital/ (68147)

6 exp Patient Care Team/ (72215)

7 Rehabilitation Centers/ (8576)

8 Inpatients/ (27507)

9 ((acute or sub-acute or subacute) adj3 (care or ward?)).tw,kf. (38212)

10 ((rehabilitation or geriatric) adj (ward? or unit? or department?)).tw,kf. (7341)

11 (acute adj5 patient?).tw,kf. (245691)

12 inpatient?.tw,kf. (130036)

13 hospital*.tw,kf. (1525967)

14 or/1-13 [hospital] (2001274)

15 Accidental Falls/pc [Prevention & Control] (10342)

16 (fall? adj2 (assess* or risk? or prevent* or reduc* or occur* or frequen* or screen*)).tw,kf. (19084)

17 15 or 16 [Falls] (23286)

18 implement*.tw,kf. (628881)

19 (sustain* adj4 (program* or practice* or intervention?)).tw,kf. (13120)

20 Adopt*.tw,kf. /freq=2 (42809)

21 Adopt*.ti. (18039)

22 deliver*.tw,kf. /freq=2 (288133)

23 deliver*.ti. (150863)

24 (adher* or comply or complian*).tw,kf. (377119)

25 fidelity.tw,kf. (34472)

26 Health Plan Implementation/ (6629)

27 implementation science/ or technology transfer/ (3267)

28 Guideline Adherence/ (34788)

29 engage*.tw,kf. /freq=2 (47833)

30 engage*.ti. (20598)

31 (intervention adj2 deliver*).tw,kf. (6761)

32 (Cognitive adj2 participat*).tw,kf. (627)

33 "buy in".tw,kf. (2105)

34 or/18-33 [Implementation or Adherence to Guidelines and strategies] (1419513)

35 Risk Assessment/mt [Methods] (38091)

36 (assess* adj4 tool?).tw,kf. (74557)

37 (electronic adj2 record?).tw,kf. (53541)

38 medical informatics/ or health information exchange/ or exp medical informatics applications/ or exp medical informatics computing/ or nursing informatics/ (490892)

39 exp Hospital Information Systems/ (30283)

40 exp health information management/ (1943)

41 exp Medical Records Systems, Computerized/ (46872)

42 (information adj3 (technolog* or system or computeri* or electronic)).tw,kf. (59321)

43 digital*.tw,kf. (181775)

44 ("risk assess*" adj4 (bundle* or instrument* or care plan*)).tw,kf. (525)

45 clinic* decision* support system*.tw,kf. (2939)

46 (electronic* adj3 nurs* adj3 document*).tw,kf. (123)

47 Decision Support Systems, Clinical/ (9161)

48 or/35-47 [Assessment tools including health IT] (868790)

49 workflow/ (8215)

50 systems integration/ (9627)

51 facilitation.tw,kf. (32835)

52 (workflow* or work-flow*).tw,kf. (37281)

53 system? fit*.tw,kf. (368)

54 Embed*.tw,kf. (153897)

55 integrat*.tw,kf. (644447)

56 (routine* or routini*).tw,kf. (440520)

57 or/49-56 [Workflows] (1271193)

58 14 and 17 and 34 and 48 and 57 [Facilitation Tools CMO 4] (38)

**Web of Science Core Collection**

Databases available at the University of Leeds (searched simultaneously):

- Arts & Humanities Citation Index (Web of Science) 1975-present
- Conference Proceedings Citation Index- Science ( Web of Science) 1990-present
- Conference Proceedings Citation Index- Social Science & Humanities (Web of Science) 1990-present
- Science Citation Index-Expanded (Web of Science) 1900-present
- Social Sciences Citation Index (Web of Science) 1900-present
- Emerging Sources Citation Index (Web of Science) 2015-present

**Search Date: 01/08/2022**

**Records found: 38**

Indexes=SCI-EXPANDED, SSCI, A&HCI, CPCI-S, CPCI-SSH, ESCI Timespan=1900-2021

# 24 #23 AND #22 AND #10 AND #6 AND #5

# 23 TOPIC: (facilitation or workflow* or work-flow* or embed* or integrat* or routine* or routini* or "system* fit*")

# 22 #21 OR #20 OR #19 OR #18 OR #17 OR #16 OR #15 OR #14 OR #13 OR #12 OR #11

# 21 TOPIC: (electronic* near/3 nurs* near/3 document*)

# 20 TOPIC: ("health information management")

# 19 TOPIC: ("clinic* decision* support system*")

# 18 TOPIC: (risk* near/3 screen* near/3 tool*)

# 17 TOPIC: ((multi-factorial or multifactorial) near/4 risk assessment*)

# 16 TS=("risk assess*" near/4 ("care plan*") )

# 15 TS=("risk assess*" near/4 (bundle* or instrument*) )

# 14 TOPIC: (digital*)

# 13 TOPIC: (electronic near/2 record$)

# 12 TOPIC: (information near/3 (technolog* or system or computeri* or electronic) )

# 11 TOPIC: (assess* near/4 tool$)

# 10 #9 OR #8 OR #7

# 9 TOPIC: (sustain* near/4 (program* or practice* or intervention$) )

# 8 TI=(adopt* or deliver* or engage*)

# 7 TS=(implement* or adher* or comply or complian* or fidelity or "buy in" or (Cognitive near/2 participat*) )

# 6 TS=(fall$ near/2 (assess* or risk$ or prevent* or reduc* or occur* or frequen* or screen*) )

# 5 #4 OR #3 OR #2 OR #1

# 4 TOPIC: (acute near/5 patient$)

# 3 TOPIC: ((rehabilitation or geriatric) near/1 (ward$ or unit$ or department$) )

# 2 TOPIC: ((acute or sub-acute or subacute) near/3 (care or ward$) )

# 1 TOPIC: (hospital* OR inpatient*)

***Patient Partnership***

**CINAHL (EBSCOhost)**

**Search Date: 01/08/2022**

**Records found: 38**

**# Query Results**

S37 S12 AND S15 AND S20 AND S29 AND S36 38

S36 S30 OR S31 OR S32 OR S33 OR S34 OR S35 922,896

S35 TI ( (compassion* or listen* or respect* or empower* or communicat*) ) OR AB ( (compassion* or listen* or respect* or empower* or communicat*) ) 647,264

S34 (MH "Empowerment") 15,895

S33 (MH "Empathy") OR (MH "Respect") 15,919

S32 (MH "Interpersonal Relations+") 312,612

S31 (MH "Listening") 4,156

S30 (MH "Compassion") 2,855

S29 S21 OR S22 OR S23 OR S24 OR S25 OR S26 OR S27 OR S28 209,365

S28 (MH "Patient Autonomy") 7,103

S27 TI ( ((person-centred or person-centered) n2 care) ) OR AB ( ((person-centred or person-centered) n2 care) ) 3,506

S26 TI ( (multifacet* or multi-facet* or tailor*) ) OR AB ( (multifacet* or multi-facet* or tailor*) ) 46,302

S25 TI ( (engag* n3 (patient? or famil* or carer? or caregiver? or player*)) ) OR AB ( (engag* n3 (patient? or famil* or carer? or caregiver? or player*)) ) 11,834

S24 (MH "Consumer Participation") 23,040

S23 TI ( (patient or patients) n5 (need* or perspective* or autonom*) ) OR AB ( (patient or patients) n5 (need* or perspective* or autonom*) ) 92,545

S22 TI ( ((patient or patients) n2 (centred or centered)) ) OR AB ( ((patient or patients) n2 (centred or centered)) ) 19,014

S21 (MH "Patient Centered Care") OR (MH "Shared Medical Appointments") 34,229

S20 S16 OR S17 OR S18 OR S19 273,020

S19 TI ( (risk n2 (assess* or evaluat*)) or guideline* or protocol* ) AND AB ( (risk n2 (assess* or evaluat*)) or guideline* or protocol* ) 39,055

S18 (MH "Protocols+") 43,946

S17 (MH "Practice Guidelines") 84,876

S16 (MH "Risk Assessment") 133,363

S15 S13 OR S14 20,168

S14 TI (fall* n2 (assess* or risk* or prevent* or reduc* or occur* or frequenc* or screen)) OR AB (fall* n2 (assess* or risk* or prevent* or reduc* or occur* or frequenc* or screen)) 15,519

S13 (MH "Accidental Falls/PC") 10,958

S12 S1 OR S2 OR S3 OR S4 OR S5 OR S6 OR S7 OR S8 OR S9 OR S10 OR S11 737,031

S11 (MH "Multidisciplinary Care Team+") 49,799

S10 (MH "Medical Staff, Hospital+") OR (MH "Nursing Staff, Hospital") 29,619

S9 (MH "Hospitals+") 128,590

S8 (MH "Hospital Units") OR (MH "Rehabilitation Centers") 16,781

S7 (MH "Inpatients") 85,946

S6 (MH "Subacute Care") OR (MH "Acute Care") 11,259

S5 (MH "Hospitalization") 43,860

S4 TI hospital* OR AB hospital* 532,701

S3 TI ( inpatient or inpatients) OR AB ( inpatient or inpatients) 61,118

S2 TI ( ((rehabilitation or geriatric) n1 (ward? or unit? or department?)) ) OR AB ( ((rehabilitation or geriatric) n1 (ward? or unit? or department?)) ) 5,417

S1 TI ( ((acute or sub-acute or subacute) n3 (care or ward?)) ) OR AB ( ((acute or sub-acute or subacute) n3 (care or ward?)) ) 29,494

**Embase Classic+Embase <1947 to 2022 July 29>**

**Search Date: 01/08/2022**

**Records found: 51**

Search Strategy:

--------------------------------------------------------------------------------

1 hospitalization/ (478011)

2 hospital department/ or exp ward/ (500590)

3 exp hospital/ (1406920)

4 medical staff/ (42122)

5 nursing staff/ (76712)

6 rehabilitation center/ (18901)

7 subacute care/ (1508)

8 exp clinical handover/ or collaborative care team/ or exp rapid response team/ (13877)

9 exp hospital patient/ (222261)

10 ((acute or sub-acute or subacute) adj3 (care or ward?)).tw,kw. (54304)

11 ((rehabilitation or geriatric) adj (ward? or unit? or department?)).tw,kw. (13043)

12 inpatient?.tw,kw. (220506)

13 (acute adj5 patient?).tw,kw. (397349)

14 hospital*.tw,kw. (2403565)

15 or/1-14 [hospital] (3569217)

16 *falling/ (13345)

17 falling/pc (2951)

18 (fall? adj2 (assess* or risk? or prevent* or reduc* or occur* or frequen* or screen*)).tw,kw. (28030)

19 or/16-18 [Falls prevention or risk assessment] (35283)

20 risk assessment/ (676072)

21 (risk adj2 (assess* or evaluat*)).tw,kw. (207299)

22 exp practice guideline/ (654361)

23 guideline*.tw,kw. (678665)

24 exp clinical protocol/ (113463)

25 protocol?.tw,kw. /freq=2 (203598)

26 or/20-25 [Multifactorial risk assessment] (1900128)

27 patient care/ (336364)

28 shared medical appointment/ (223)

29 (patient? adj2 (centred or centered)).tw,kw. (38317)

30 (patient? adj5 perspective?).tw,kw. (36791)

31 (patient? adj4 need?).tw,kw. (134309)

32 patient participation/ (32642)

33 (engag* adj3 (patient? or famil* or carer? or caregiver? or player*)).tw,kw. (22707)

34 (multifacet* or multi-facet* or tailor*).tw,kw. (186436)

35 ((person-centred or person-centered) adj2 care).tw,kw. (3687)

36 patient autonomy/ (6808)

37 (patient? adj3 autonom*).tw,kw. (12599)

38 ((individuali* or personali*) adj2 care).tw,kw. (10122)

39 or/27-38 [New Patient Centred Care] (750341)

40 empathy/ (30690)

41 compassion*.tw,kw. (18236)

42 exp interpersonal communication/ (767566)

43 listen*.tw,kw. (45751)

44 respect/ (1815)

45 (empower* adj3 (staff or professional? or nurs* or doctor? or clinician? or patient?)).tw,kw. (10670)

46 empowerment/ (11880)

47 respect*.tw,kw. /freq=2 (1083331)

48 respect*.ti. (14558)

49 communicat*.tw,kw. /freq=2 (125774)

50 communicat*.ti. (89269)

51 or/40-50 [Compassion Empowerment] (2018728)

52 15 and 19 and 26 and 39 and 51 [Falls in Hospital and Risk Assessment and Person-centred care and Empowerment Compassion] (51)

**Ovid MEDLINE(R) ALL <1946 to July 29, 2022>**

**Search Date: 01/08/2022**

**Records found: 20**

Search Strategy:

--------------------------------------------------------------------------------

1 Hospitalization/ (129166)

2 Subacute Care/ (1351)

3 Hospital Units/ (10446)

4 exp Hospitals/ (306482)

5 medical staff, hospital/ or nursing staff, hospital/ (68147)

6 exp Patient Care Team/ (72215)

7 Rehabilitation Centers/ (8576)

8 Inpatients/ (27507)

9 ((acute or sub-acute or subacute) adj3 (care or ward?)).tw,kf. (38212)

10 ((rehabilitation or geriatric) adj (ward? or unit? or department?)).tw,kf. (7341)

11 (acute adj5 patient?).tw,kf. (245691)

12 inpatient?.tw,kf. (130036)

13 hospital*.tw,kf. (1525967)

14 or/1-13 [hospital] (2001274)

15 Accidental Falls/pc [Prevention & Control] (10342)

16 (fall? adj2 (assess* or risk? or prevent* or reduc* or occur* or frequen* or screen*)).tw,kf. (19084)

17 15 or 16 [Falls] (23286)

18 exp Risk Assessment/ (304882)

19 (risk adj2 (assess* or evaluat*)).tw,kf. (158055)

20 guideline/ or practice guideline/ (37170)

21 guideline*.tw,kf. (436990)

22 protocol?.tw,kf. /freq=2 (144789)

23 protocol?.ti. (82808)

24 exp Clinical Protocols/ (185718)

25 or/18-24 [Multifactorial risk assessment] (1170591)

26 exp Patient-Centered Care/ (23405)

27 Shared Medical Appointments/ (69)

28 (patient? adj2 (centred or centered)).tw,kf. (30442)

29 (patient? adj5 perspective?).tw,kf. (25542)

30 (patient? adj4 need?).tw,kf. (81555)

31 Patient Participation/ (28786)

32 (engag* adj3 (patient? or famil* or carer? or caregiver? or player*)).tw,kf. (15786)

33 (multifacet* or multi-facet* or tailor*).tw,kf. (149582)

34 (person adj2 (centred or centered)).tw,kf. (8428)

35 personal autonomy/ (18073)

36 autonom*.tw,kf. (160104)

37 ((individuali* or personali*) adj2 care).tw,kf. (7146)

38 or/26-37 [Patient Centred Care] (499191)

39 Empathy/ (21941)

40 compassion*.tw,kf. (13607)

41 listen*.tw,kf. (38347)

42 exp Interpersonal Relations/ (346375)

43 respect*.tw,kf. /freq=2 (718155)

44 respect*.ti. (11900)

45 respect/ (786)

46 empower*.tw,kf. (34326)

47 empowerment/ (688)

48 communicat*.tw,kf. /freq=2 (103219)

49 communicat*.ti. (74092)

50 or/39-49 [Compassion Empowerment] (1263366)

51 14 and 17 and 25 and 38 and 50 [Falls in Hospital and Risk Assessment and Person-centred care and Empowerment Compassion] (20)

**Web of Science Core Collection**

Databases available at the University of Leeds (searched simultaneously):

- Arts & Humanities Citation Index (Web of Science) 1975-present
- Conference Proceedings Citation Index- Science ( Web of Science) 1990-present
- Conference Proceedings Citation Index- Social Science & Humanities (Web of Science) 1990-present
- Science Citation Index-Expanded (Web of Science) 1900-present
- Social Sciences Citation Index (Web of Science) 1900-present
- Emerging Sources Citation Index (Web of Science) 2015-present

**Search Date: 01/08/2022**

**Records found: 32**

Indexes=SCI-EXPANDED, SSCI, A&HCI, CPCI-S, CPCI-SSH, ESCI Timespan=1900-2021

# 19 #18 AND #17 AND #9 AND #6 AND #5

# 18 TOPIC: (compassion* or listen* or respect* or empower* or communicat*)

# 17 #16 OR #15 OR #14 OR #13 OR #12 OR #11 OR #10

# 16 TOPIC: (patient$ near/3 autonom*)

# 15 TS=((person-centred or person-centered or individuali* or personali*) near/2 care)

# 14 TOPIC: (multifacet* or multi-facet* or tailor*)

# 13 TOPIC: (engag* near/3 (patient$ or famil* or carer$ or caregiver$ or player*) )

# 12 TOPIC: (patient$ near/4 need$)

# 11 TOPIC: (patient$ near/5 perspective$)

# 10 TOPIC: (patient$ near/2 (centred or centered) )

# 9 #8 OR #7

# 8 TOPIC: (guideline* or protocol*)

# 7 TOPIC: (risk near/2 (assess* or evaluat*) )

# 6 TOPIC: (fall$ near/2 (assess* or risk$ or prevent* or reduc* or occur* or frequenc* or screen) )

# 5 #4 OR #3 OR #2 OR #1

# 4 TOPIC: (acute near/5 patient$)

# 3 TOPIC: ((rehabilitation or geriatric) near/1 (ward$ or unit$ or department$) )

# 2 TOPIC: ((acute or sub-acute or subacute) near/3 (care or ward$) )

# 1 TOPIC: (hospital* OR inpatient*)
