# Supplementary material for: Exploring variation in implementation of multifactorial falls risk assessment and tailored interventions: a realist review
Source: BMC Geriatr. 2023 Jun 21;23:381. doi: 10.1186/s12877-023-04045-3 (PMC10286425; doi:10.1186/s12877-023-04045-3)
Supplement: Supplementary file 3 — Supplementary Material 3 [file 12877_2023_4045_MOESM3_ESM.docx]

Appendix iii: Study details

| **Facilitation** | | | | |
| --- | --- | --- | --- | --- |
| **Citation & country** | **Setting** | **Sample** | **Intervention Description** | **Study Design (inc. comparator if applicable)** |
| 1. Albornos-Munoz et al. 2018, Spain | Two medical and one surgical ward in one hospital. | Patients of 65 years or over. | Multi-faceted fall prevention strategy with patient-level, nurse-level, and unit-level interventions used to overcome barriers to implementation. | Quality improvement project, clinical audit conducted pre-post intervention. |
| 2. Businger et al., 2020, US | Twelve inpatient units in an acute care hospital. | Stakeholders including study personnel. Tools impacted 12,628 patient admissions | The Patient Safety Learning Laboratory, a suite of Health Information Technology tools integrated within an Electronic Health Record vendor system. | Observational study, to share challenges, recommendations and lessons learned from implementation. No comparator. |
| 3. Capan & Lynch, 2007, US | 357 bed acute hospital. | Patients admitted to the hospital. | Multifaceted fall prevention strategy with patient-level, nurse-level, and unit-level interventions, including site specific fall risk assessment and intervention tool that underwent pilot testing. | Quality improvement project, compared rate of falls before and after introduction of strategy. |
| 4. Carroll et al., 2012, US | Four hospitals (two academic medical centers and two community hospitals) within a single healthcare system. | Randomly selected medical records for patients on the eight study units (four intervention units; 5,267 patients) and four usual care units (5,116 patients) during three separate study visits. | Fall TIPS (Tailoring Interventions for Patient Safety) toolkit, integrated in electronic health records. Staff entered risk data, and the software tailored fall prevention interventions to address specific determinants of falls risk. The toolkit generated bed posters comprising brief text with accompanying icons, patient education hand-outs, and plans of care. | Nursing documentation related to fall risk and prevention was reviewed to evaluate the effectiveness of the toolkit for promoting documentation of fall risk status and planned and completed fall prevention interventions. Comparator was documentation in the usual care units. |
| 5. Cook et al., 2020, US | Trauma centre, emergency department in a tertiary care teaching hospital. | Adult patients. | Multifaceted fall prevention strategy with patient-level, nurse-level, and unit-level interventions including an ED-specific fall risk assessment tool. | Quality improvement project. Comparator: post-intervention monthly unit data. |
| 6. Currie et al., 2006, US | A large multi-site academic medical centre. | 25% sample of patients in fifty-two nursing units at the three sites. | Fall-Injury Risk Assessment instrument integrated into three different platforms. | Bedside audits were performed every 5 weeks. Compliance with use of the instrument in units in which it was integrated into the  daily shift assessment was compared with units that did not  have complete electronic nursing documentation. |
| 7. Dempsey, 2004, Australia | Acute medical wards in a regional teaching hospital. | Patients admitted in two periods (pre-post intervention). | Multifaceted fall prevention strategy with patient-level, nurse-level, and unit-level interventions including a site-specific falls risk assessment tool and a choice of interventions. | Quality improvement project - practice review after 5 years of falls prevention programme to assess sustainability. Comparator: two cohorts of patients (pre-post intervention). |
| 8. Dowding et al., 2012, US | Twenty-nine hospitals in a large integrated health care organization. | Patient clinical records. | Integrated EHR including computerized physician order entry, nursing documentation, risk assessment tools, and documentation tools. | Interrupted time series analysis examining hospital acquired pressure ulcers (HAPUs) and falls. Comparator: % patients with  completed risk assessments for  HAPUs/falls and rates of HAPUs/falls compared pre- and post-EHR implementation. |
| 9. Duckworth et al., 2019, US | Six Neurology units and seven medical or medical-surgical units in three acute care centres. | Nurses submitted 1209 audits for the patient engagement measure and 1401 for the presence of the Fall TIPS poster at the bedside. | Fall TIPS in three modalities including: (1) laminated Fall TIPS poster; (2) electronic Fall TIPS poster; and (3) paperless patient safety e-bedside display. | Implementation science study auditing patient engagement and adherence with poster display. Compared modalities of intervention. |
| 10. Dykes et al., 2009, US | Four acute care hospitals. | 685 patients on the units using the Fall TIPS toolkit. | Original Fall TIPS toolkit, which identified a core set of evidence-based interventions directly linked to the patient-specific risk factors and generated a bed poster, a plan of care and an educational handout. | Interim paper assessing impact of adoption strategies *via* audits. Compared mean number of fall risk assessments completed  by nurses using Fall TIPS per patient, per day from first month after implementation to present. |
| 11. Dykes et al., 2017, US | Two large medical centres. | 31 patients on the medical units answered the pre surveys, and 33 patients answered the post surveys. | Fall TIPS: Framework for Spread was used to support uptake and included four phases: (1) communicating ‘better ideas’; (2) planning and setup; (3) spread within the target population; and (4) continuous monitoring and feedback related to adoption and spread of the innovation. | Quality improvement project involving patient surveys and measuring protocol adherence, patient falls and falls-related injury rates. No comparator. |
| 12. Healey et al., 2004, UK | Care of older person wards and associated community units of a district general hospital. | Control (956 pre, 905 post); Intervention (776 pre, 749 post). | In the intervention wards, staff used a pre-printed care plan for patients identified as at risk of falling and introduced appropriate remedial measures. | A group (ward) randomised trial evaluated impact on relative risk of falls. Comparator: usual care on control wards. |
| 13. Hefner, et al., 2015, US | A large health system, comprising five hospitals. | 800 spot checks of patient rooms. | Falls Wheel (visual tool): the top circle instructed viewers that universal fall precautions should be implemented for all patients; information about the specific fall risk categories then guided which additional safety measures were to be put into place for each patient. | Quality improvement project, pre-post intervention audits. |
| 14. Ireland et al. 2010, Canada | Sixty units in a Registered Nurses’ Association of Ontario (RNAO) healthcare organisation. | Random audits of 193 patient medical records on 15 medical-surgical units were undertaken. | Hospital-wide, multifaceted fall prevention strategy with patient-level, nurse-level, and unit-level interventions strategy called 'Don't Fall for It', comprising routine patient risk screening of *all* patients and universal and targeted interventions. | Quality improvement project, pre-post intervention audits. |
| 15. Koh et al., 2008, Singapore | Medical, surgical, geriatric units, at five acute care hospitals. | Nurses (n = 1830). | N/A | Survey to identify nurse perceptions of barriers to implementing a clinical practice guideline. The validated questionnaire, 'Barriers and facilitators assessment instrument', was administered. No comparator. |
| 16. Koh et al., 2009, Singapore | Two acute care hospitals with closely matched perceived barriers to implementation of innovation. | All nursing staff (n = 641) working in medical, surgical and geriatric units. 193 patient records on 15 medical-surgical units. | Multifaceted fall prevention strategy with patient-level, nurse-level, and unit-level interventions designed to address barriers to implementation and including use of a mandatory falls risk assessment tool. | Comparative study, pre-intervention, post intervention and six-month follow-up including knowledge assessment of nursing staff, and audits of fall rates and fall prevention practices. |
| 17. Lytle et al., 2015, US | Sixteen adult units (general medicine and surgical) in an academic health centre. | One medical and one surgical unit were selected for retrospective chart review. | Falls prevention Computer Decision Support tools including reminders for: (1) ‘admission documentation incomplete’; (2) ‘shift documentation incomplete’; and (3) a ‘rules-based alert’ for patients at high risk of falls and not on a fall prevention plan of care. | Quality improvement project, which used a pre/post quasi-experimental study design. |
| 18. Maia et al., 2018, Brazil | Two units in a University Hospital. | Adult and older adult inpatients (48 in one unit, 18 in the other). | Multi-faceted fall prevention strategy with patient-level, nurse-level, and unit-level interventions, to overcome barriers to implementation. Included introduction of standardised falls risk assessment tool. | Quality improvement project - clinical audit conducted pre-post intervention. |
| 19. McCarty et al., 2018, US | Emergency departments (12 sites) of an integrated health care delivery system | Eleven of the 12 ED’s were visited over a 4-month period; 60 nurses attended training sessions. | Multi-faceted fall prevention strategy with patient-level, nurse-level, and unit-level interventions including introduction of the Emergency Department Fall-Risk Assessment Tool (MEDFRAT), programmed into the EHR. | Quality improvement project focused on implementation of strategy. No comparator. |
| 20. Milisen et al., 2013, Belgium | Seventeen geriatric wards, selected at random out of 40 hospitals. | Forty-nine healthcare workers. | A practice guideline including four consecutive parts: (1) case finding, i.e., identification of persons at risk for falling; (2) in-depth multifactorial assessment of risk factors; (3) targeted interventions; and (4) transfer of information at discharge. | Feasibility study using questionnaire. No comparator. |
| 21. Ohde et al., 2012, Tokyo | A 520-bed community-based, tertiary-level, teaching hospital. | All adult inpatients, except for maternity, preventative health screening and intensive care patients. | Multi-faceted fall prevention strategy with patient-level, nurse-level, and unit-level interventions including introduction of a fall risk assessment tool and intervention protocol. | Quality improvement project. Within group pre-post intervention comparison. |
| 22. Pop et al., 2020, US | Emergency Department academic medical centre. | Staff participating in education sessions. | Components of the intervention were selected on the basis of a review of fall prevention research and included fall risk assessment, safe ambulation, safe toileting, staff communication, early warning, and patient education. | Quality improvement project. No comparator. |
| 23. Teh et al., 2017, Australia | Acute Medical Unit and Geriatric Evaluation and Management (GEM) unit at a tertiary teaching hospital. | All patients admitted to both wards during the trial period. (AMU n = 424, GEM n = 111). | iPad^TM^-based assessment tool. Black-and-white A4-sized bedside posters were automatically printed at assessment completion to be displayed at patient’s bedside. | Trial comparing the iPad-based tool with the traditional Fall Risk for Older Person (FROP) tool in fall risk screening. |
| 24. Teh et al., 2018, Australia | Acute Medical Unit and Geriatric Evaluation and Management (GEM) unit at a tertiary teaching hospital. | Ward staff - pre-trial focus group n=5, survey n=48; post trial focus group n=5, survey n=29. | iPad^TM^-based assessment tool for direct clinician entry of up to 13 common falls risk activities, with automatic generation of visual cues for bedside display. | Mixed methods pilot study with focus groups and surveys. Pre- and post-trial focus findings were compared. |
| 25. Thatphet et al., 2021, US and Canada | Five hospitals EDs located in the US and Canada. | Three physicians, two ED nurses, and one program coordinator (key informants). | N/A: describes the experiences of emergency departments with geriatric fall programs. | Semi-structured, open-ended telephone/skype interviews recruited from a purposeful sampling technique. No comparator. |
| 26. Titler et al., 2016, US | Thirteen adult medical-surgical units from three community hospitals. | Licensed nurses (n=157 pre; 140 post) and adult patients (n=390 pre and post). | Targeted Risk Factor Fall Prevention Bundle, focusing on interventions that reduced or modified patient-specific fall risk factors. A Translating Research Into Practice (TRIP) multifaceted implementation intervention was used to promote uptake and use of the fall prevention bundle. | A prospective pre–post implementation cohort design using questionnaires (Stage of Adoption, and Use of Research Findings in Practice Scale). |
| 27. Townsend et al., 2016, US | Emergency Department in one hospital. | Reviewed fall data for each quarter of 2013, including risk assessments scores, the total number of falls, and the circumstances of each fall. | Multi-faceted fall prevention strategy with patient-level, nurse-level, and unit-level interventions including introduction of an emergency department-specific fall risk tool, the KINDER1. | Quality improvement project. Data were collected and compared retrospectively prior to the project launch and concurrently after project implementation. |
| 28. Wu et al., 2019, Taiwan | One medical centre. | Clinical records of 19695 patients were analysed. | Standardized Computerized Nursing Process Documentation System, including electronic data entry, scores on the risk assessment template and health assessment and Nursing Care Plans. | Retrospective study to identify patients at high risk of falls and explore the relationship between Nursing Care Plans and falls and pressure injury incidences. A run chart of fall injury incidence rate from 2007 to 2017 used  to determine effect of SCNPDS. |

| **Patient Participation** | | | | |
| --- | --- | --- | --- | --- |
| **Citation & country** | **Setting** | **Sample** | **Intervention Description** | **Study Design (inc. comparator if applicable)** |
| 1. Bargmann & Brundrett, 2020, US | A medical-surgical telemetry unit in a military trauma centre. | Initial post- implementation audit was undertaken with 17 patients and project began once 90% of staff received face-to-face training. | Multi-faceted falls prevention bundle, including (1) daily patient education on what contributed to their falls risk during shift assessments; (2) patient educational hand-out on fall risk factors; and (3) a fall safety agreement, which patients were encouraged to sign. | Quality improvement project, compared baseline and post-intervention falls rates per 1000 patient days. |
| 2. Cann & Gardner, 2012, Australia | Acute surgical ward in a hospital. | All adult patients and ward nursing staff. 1115 patients admitted pre-implementation and 1069 post-implementation. | Practice Partnership Model of Care with four components: (1) staff working in partnership with each other, rather than each nurse providing exclusive care for an individual caseload of patients; (2) clinical handover at the bedside; (3) comfort rounds every 1-2 hours; and (4) environmental modifications. | Quality improvement project: Pre-test—post-test. |
| 3.Carroll et al., 2010, US | An acute care hospital. | Nine patients who had fallen while inpatients, within 48 hours of interviews. | N/A: patients were interviewed about their experiences of a fall and how further falls could be prevented. | Qualitative interviews, no comparator. |
| 4. Christiansen et al., 2020, US | Medical units at three acute hospitals. | Patients (N = 343) | Fall TIPS (Tailoring Interventions for Patient Safety), available in three modalities: (1) laminated poster; (2) electronic poster; (3) patient safety e-bedside display. Authors stated that each unit selected the modality that worked best for that unit’s workflow, but did not report which units chose which modality, or report results per modality. | Patient survey. The short form Patient Activation Measure (PAM-13) adapted for fall prevention assessed patient’s knowledge, skill, and confidence in managing his or her fall prevention. Comparator: patient activation measured before and after implementation of Fall TIPS in the three hospitals. |
| 5. Duckworth et al., 2019, US | Six Neurology units and seven medical or medical-surgical units in three acute care centres. | Nurses submitted 1209 audits for the patient engagement measure and 1401 for the presence of the Fall TIPS poster at the bedside. | Fall TIPS: study examined whether three modalities of Fall TIPS (original EHR version; a laminated poster version; and e-bedside display version) impacted on patient engagement in falls prevention process and thus on Fall TIPS efficacy. | Implementation science study auditing patient engagement and adherence with poster display. Compared modalities of intervention. |
| 6.Dykes et al., 2010, US | Four acute hospitals in one healthcare system. | All patients admitted to the hospitals during study period. 5160 patients in intervention group, and 5104 patients in control group. | Fall TIPS: original Fall TIPS toolkit, a tool within participating hospitals’ electronic health records, which integrated existing communication and workflow patterns. Once staff entered risk data from patients, the software tailored fall prevention interventions to address specific determinants of falls risk. The toolkit produced bed posters composed of brief text with an accompanying icon, patient education handouts, and plans of care. | Quantitative stratified, cluster randomised trial. Comparator: four control units in the four participating hospitals, where patients received usual care. |
| 7. Dykes et al., 2017, US | Two oncology, three neurology, and two medical units at one hospital, and a large medical unit at a second hospital. | At the first hospital, 31 patients on the medical units answered pre-surveys, and 33 patients answered post-surveys. At the second hospital 32 patients answered pre surveys, and 30 patients answered post-surveys. | Fall TIPS: a development of the Fall TIPS intervention, intended to enhance its patient-centred focus. In this iteration, a low-tech modality was added to the original EHR-based tool, in the form of a colour-coded, icon-based laminated paper poster in English and Spanish. | Quality improvement project involving patient surveys and measuring protocol adherence, patient falls and falls-related injury rates. No comparator. |
| 8. Dykes et al., 2020, US | Fourteen adult medical units in three academic medical centres. | All patients admitted to participating units during study period, comprising: 17,948 pre-intervention and 19,283 post-intervention. | Fall TIPS: three modalities of the Fall TIPS toolkit: (1) original EHR-based tool; (2) care plan displayed on laminated paper poster; and (3) care plan displayed on electronic e-bedside screen display. | Quantitative non-randomised control trial. Each unit served as its own control. |
| 9. Goldsack et al., 2015, US | An adult medical stroke unit (Unit 1) and a haematology/ oncology unit (Unit 2) in a hospital. | Fifty-six patient flow sheets selected for review: 27 from Unit 1 and 29 from Unit 2.  One hundred and eight staff were surveyed about the last round they completed. Twenty staff in Unit 1 and 20 staff in Unit 2 also took part in a post-implementation survey. | Patient-centred proactive hourly rounding, conducted every hour between 0600 and 2200 hours and every 2 hours between 2200 and 0600 hours. Rounding was performed by nurses and patient care technicians (Unit 1) or nurses only (Unit 2) based on differences in registered nurse staffing between the two units. | Quality improvement project. Patient falls rates per 1000 patient days were compared on the two participating units before and after implementation. |
| 10. Haines et al., 2011, Australia | Acute (orthopaedic, respiratory, & medical) and subacute (geriatric, neurorehabilitation) wards in two hospitals. | Older hospital patients (> 60 years), n=1206: 401 patients in the complete program group, 424 in the materials only group, and 381 in the control group. | Multimedia patient education program combined with trained health professional follow-up (complete program), multimedia patient education materials alone (materials only), and usual care (control). | Three group randomised control trial. Comparator: participants randomised to the control group, who received usual care. |
| 11. Hill et al., 2015, Australia | Eight aged care rehabilitation hospital units | All physiotherapists (N=10) trained as ‘educators’ in delivering the Safe Recovery program were invited to participate in the focus group. | Safe Recovery Program: individualised education which involved providing patients with a multimedia package (a DVD to view and a written workbook to read) followed up by between one and three individualised sessions with a physiotherapist trained as an educator. Educators also trained multidisciplinary staff to facilitate their support of the programme. | A qualitative exploratory study including a focus group and an interview (n=10 educators), and review of written educator notes and reflective researcher field notes based on interactions with the educators during the primary study. No comparator. |
| 12. Hill et al., 2016, Australia | Eight aged care rehabilitation hospital wards in hospitals that provide acute and rehabilitation care. | Participants who responded (n=473) Older patients (n=757) who were eligible (mini-mental state examination score>23/30) | Safe Recovery Program: The education programme provided participants with a three-step message: (1) know if you need help; (2) ask for help; and (3) wait for help. The educator helped participants to develop a personalised action plan consisting of strategies that allowed them to engage safely in required mobility tasks on the ward and work cooperatively with staff, such as ringing the bell if they required help. | A prospective qualitative survey to understand patient response to the program and their identified barriers to engaging in falls prevention strategies. No comparator. |
| 13. Johnson et al., 2011, US | Nursing department in one hospital. | All patients admitted to the hospital in study period: 12,159 patients were assessed over three years (2,231 in 2008; 4,739 in 2009; and 5,189 in 2010). | Multi-faceted falls prevention programme, called ‘Helping Hands’, which including engaging patients and families in falls prevention and asking them to sign a fall safety agreement contract. | Quality improvement project, compared rate of falls pre- and post-implementation. |
| 14. Kiyoshi-Teo et al., 2019, US | Three medical-surgical units at a Veterans Health Administration (VA) hospital. | Sixty-seven older in patients (> 65 years). | N/A: reports older inpatients’ responses to surveys about the level of importance and confidence they felt for fall prevention in their current hospitalised state; their fears or concerns about falling; their levels of activation, willingness and ability to make independent actions to manage their health and care; daily activities they undertook to prevent themselves from falling; risk of falls due to medications; and cognitive status. | Quantitative surveys and chart reviews. No comparator. |
| 15. Kullberg et al., 2015, Sweden | Department of Oncology in one hospital. | One hundred and four cancer patients. | N/A: reports patients' perceptions of information exchange about falls prevention with doctors and nurses and associations with patient satisfaction, participation and safety at inpatient oncology wards. | Quantitative questionnaires. No comparator. |
| 16. Martin et al., 2020, New Zealand | Four rehabilitation wards in an older person's health rehabilitation hospital. | Seventy-two patients took part in surveys. Forty-nine staff took part in pre-intervention surveys and 44 in post-intervention surveys.  Patients took part in qualitative interviews (n = 11). Eight ward staff took part in focus groups, along with six Safe Recovery Programme educators. | Safe Recovery Programme: used patient-directed education and individualised goal setting to educate patients about how to keep themselves safe in hospital. Two SRP educators (1.4 FTE), a physiotherapist and a nurse, were employed to implement the pilot. A3 wall posters were used to highlight key SRP messages as well as recording individual goals patients had identified to keep themselves safe. Four retired nurse volunteers delivered initial SRP education and goal-setting sessions to individual patients. | Realist evaluation, involving qualitative surveys, interviews and focus groups. No comparator. |
| 17. Radecki et al., 2018, US | An academic health centre. | Twelve patients. | N/A: reports patients’ perspectives of falls prevention in an acute care setting, to aid in the design of patient-centred strategies. | Qualitative interviews. No comparator. |
| 18. Radecki et al., 2020, US | Four non–intensive care inpatient units in a trauma centre. | Two hundred and three patients (103 at baseline and 100 during the intervention) completed knowledge-in-action survey. Forty nurses completed a nurse usability survey. | Patient Fall self-Assessment Tool (PFAT): a self-completed assessment tool to engage patients to coproduce the fall prevention plan. Within 24 hours of admission to the unit, nurses and patients identified risk factors and developed a fall prevention plan together. The nurse transcribed the risk factors and plan onto a laminated board in the patient’s room, which included areas to record activity status and level of assistance needed for mobilisation, and to select safety equipment. | Quality improvement project using patient and staff surveys. Compared rates of falls and falls with injury per 1000 patient-days during baseline (9 weeks before implementation) and implementation. |
| 19. Rush et al., 2009. Country not stated but appears to be US | Cardiology, urogynaecology, general surgery and trauma units in a hospital. | Fifteen nurses. | N/A: reports acute care nurses’ experiences with patient falls. | Qualitative focus groups. No comparator. |
| 20. Sitzer et al., 2016, US | Four acute care units, four progressive care units and one short-stay observation unit in an acute care community hospital. | Sixty patients in the acute care units, 35 patients in the progressive care units, and 25 patients in the short-stay observation unit. | Self-assessment for falls risk (SAFR) and fall prevention education. Patients performed their own fall risk assessment via a six-item questionnaire using a modified version of the Schmid fall risk assessment tool on an interactive device in their rooms. They were notified automatically of their falls risk status and provided with additional resources to prevent falls, such as a fall prevention video. | Quality improvement project. No comparator. |
| 21. Turner et al., 2019, UK | Two rehabilitation wards in a general hospital. | Five older patients (aged in 70s and 80s) who fell on the wards. | N/A: reports experiences of older patients who fell during their hospital stay. | Qualitative interviews and document review. No comparator. |
| 22. Twibell et al., 2015, US | Acute teaching hospital. | One hundred and fifty-eight patients. | N/A: reports hospitalised adults’ perceptions related to risk for falling, fear of falling, expectations of outcomes of falling, and intention to engage in behaviours to prevent falls. | Quantitative correlational study. Nurses’ assessments and patients’ perceptions of the risk for falling were compared. |
| 23. Vonnes et al., 2017, US | Comprehensive Cancer Centre. | Patients from low to high risk were required to sign the Fall Prevention Agreement on admission. | To promote patient and family participation in the fall reduction and safety plan, the Fall Risk and Prevention Agreement was introduced upon admission. Using the Morse Fall Scoring system, patients’ risks of falling was communicated on the Fall Risk and Prevention Agreement. Besides admission, patients were reassessed based on change of status, transfer or after a fall occurred. | Quality improvement project. Compared falls and falls injuries rates two-quarters prior to implementation of the agreement and eight-quarters post-implementation. |
| 24. Zadvinskis et al., 2019, US | 41 inpatient nursing units across seven healthcare facilities in a healthcare system. | Eight hundred and eight registered nurses surveyed about their falls practices and levels of engagement. | Purposeful rounds, during which staff intentionally checked on patients at regular intervals to ensure their needs were being met. | Quantitative descriptive analysis. Compared falls rates in the nursing units but not over time. |
