# Supplementary material for: Exploring variation in implementation of multifactorial falls risk assessment and tailored interventions: a realist review
Source: BMC Geriatr. 2023 Jun 21;23:381. doi: 10.1186/s12877-023-04045-3 (PMC10286425; doi:10.1186/s12877-023-04045-3)
Supplement: Supplementary file 4 — Supplementary Material 4 [file 12877_2023_4045_MOESM4_ESM.docx]

Appendix iv: GRADE CerQual

| **Summary of review finding** | **Studies contributing to the review finding** | **Methodological limitations** | **Coherence** | **Adequacy** | **Relevance** | **CERQual statement of confidence in the evidence** | **Explanation of CERQual assessment** |
| --- | --- | --- | --- | --- | --- | --- | --- |
| Where MFRA tools are clearly visible to staff in their work routines, they can prompt delivery and documentation of a falls risk assessment. However, there is variation in practice because MFRA tools vary in number and type of assessment items and whether they stratify patients by risk. | [1-17] | **Minor to Moderate concerns:** Eleven studies were QI projects that varied in levels of detail provided about methods. Five studies used quantitative methods (including an RCT, Interrupted Time Series analysis,) had clearly described aims and methods, as did one semi-structured interview study. | **Minor to Moderate Concerns:** Three studies described tools as a practice reminder/prompt. Eleven studies measured compliance documenting MFRAs with ten demonstrating improvement and one no change pre-post tool. Three studies demonstrated that tool visibility can constrain use of tool.  The 17 studies describe use of publicly available and locally developed tools that vary in assessment items and use of stratification. | **Moderate to Serious concerns:** Data presented are largely quantitative, focusing on process measures such as compliance in documenting an assessment. Differences in assessment items can be distinguished where tools are clearly described. There is little data about staff experiences using tools but staff feedback and focus group data from two studies suggest lack of visibility can constrain tool use. | **Minor concerns:** all studies were conducted in inpatient, acute settings with adults/ older patients. Eight studies were undertaken in the US. The remaining studies took place in the US and Canada, the UK, Spain, Australia, Singapore, Brazil, Tokyo and Taiwan. | **Moderate confidence** – it is likely that the review finding is a reasonable representation of the phenomenon of interest | Minor-moderate concerns regarding methodological limitations, coherence, and relevance. |
| Documentation and delivery of processes that follow a falls risk assessment, e.g., use of targeted interventions, can be constrained by changes in patient condition, movement between wards, availability of interventions recommended in tools, and ineffective communication between different professional groups, leading to variation in practice as documented in the clinical record. | [1-3, 8-11, 15, 16, 18, 19] | **Minor to Moderate concerns:** Seven studies were QI projects that varied in levels of detail provided about methods. Four studies clearly described methods that were appropriate to answer clear research questions. | **Minor to moderate concerns:** Five papers show improvement in documentation of a falls risk assessment, but variation in documentation of follow-up processes. One paper reported that improvement was not sustained over the longer term. Eight papers discuss constraints on follow-up processes, but data to support these claims are variable. | **Moderate to Serious concerns:** Data are mostly quantitative and take the form of process measures, e.g., use of targeted interventions as documented in the clinical record. Some focus group and survey data about user experience provided in two studies. | **Minor concerns:** All studies were conducted in inpatient settings with adults and included, or focused on, older patients. Five studies took place in the US. The remaining studies took place in Australia, Belgium, Brazil, Spain, Taiwan and Tokyo. | **Moderate confidence** – it is likely that the review finding is a reasonable representation of the phenomenon of interest | Minor-moderate concerns regarding methodological limitations, coherence, and relevance. |
| Tool stratification of patients at high-risk of falls does not always align with staff clinical judgement and tool use can be constrained where tools do not provide space to document clinical judgement. | [2, 9, 15, 17] | **Moderate to**  **Serious concerns:** Three out of four studies are QI projects and vary in the amount of detail provided about methods. Some findings are anecdotal rather than linked to specific data collection methods. | **Minor concerns:** Three studies indicated that tools were not used as intended where tools did not align with clinical judgement. In one study, space to document clinical judgement was said to empower staff and support allocation of limited resources. | **Moderate to Serious concerns**: The amount and richness of data supporting explanation of clinical judgement is variable, with some limited to author accounts. | **Minor concerns**: All studies were conducted in inpatient setting, with adult/inpatient population. Three studies were conducted in the US and one in Taiwan. | **Low to Moderate confidence** – it is possible/likely that the review finding is a reasonable representation of the phenomenon of interest | Moderate/Serious concerns in methods and adequacy. |
| HIT can facilitate delivery of falls prevention practices by automating processes, removing clinicians’ task-load. However, HIT introduces additional training needs and user tasks (e.g. poster display) that may be seen as competing priorities on staff time. | [20-26] | **Minor to moderate concerns:** Five studies audited intervention compliance (two of which were QI projects) that vary in level of detail provided about research methods. One study was an observational study. One study was mixed methods pilot with clearly described research aims and objectives. | **Minor concerns:** Six studies examined technology that automated falls prevention practices, showing good levels of adherence. Three studies detailed staff experiences using the HIT and the impact of novel manual tasks, suggesting experience, training, and competing priorities may influence use at the ward level. | **Minor concerns:** Qualitive and quantitative data were reported, detailing staff experiences of tool use and compliance using HIT. | **Minor concerns**: all studies were conducted in acute inpatient settings with adults and included older patients. Six studies took place in the US and the remaining study took place in Australia. | **High confidence:** It is highly likely that the review finding is a reasonable representation of the phenomena of interest. | Minor concerns in coherence, adequacy and relevance. Minor/moderate concerns in methodological limitations. |
| In hospital, patient circumstances and beliefs can constrain their participation in falls prevention strategies. These circumstances include not wanting to disturb busy nurses by requesting their help, not perceiving or believing they are at risk of falls, not understanding their falls risks. | [27-34] | **Minor concerns:**  The eight papers included five qualitative studies, two quantitative studies and a mixed methods realist evaluation. Most studies clearly described research aims and methods. | **Minor concerns:** Seven studies discuss patient experiences with falls prevention including reasons why patients might not participate in falls prevention activities. Three studies explored nurses’ experiences of falls and interactions with patients. | **Minor concerns:** The qualitative studies provide rich data from patient and nurses’ perspectives and one realist evaluation explained the links between these experiences and appropriate ‘messaging’. | **Minor concerns:** All studies were in acute settings except one rehab hospital. Five studies took place in the US, one in Australia, one in New Zealand and one in the UK. | **High confidence:** It is highly likely that the review finding is a reasonable representation of the phenomena of interest. | Minor concerns in coherence, adequacy and relevance. |
| Where hospital staff understand patients’ circumstances through meaningful and directed interactions, they can personalise falls prevention messages to improve patient knowledge, skills and confidence to participate in falls prevention strategies. | [22, 24, 28, 30, 35-39] | **Minor concerns:** Two studies were QI, five were quantitative (including one randomised and one nonrandomised trial), two were qualitative and one a mixed methods realist evaluation. The majority clearly described research aims and methods. | **Minor - moderate concerns:** Two studies indicated improvement in patient knowledge and confidence, three studies indicated good levels of adherence to patient engagement measures, and one study indicated that the intervention helped reconcile differences in patient/staff perspectives. In one study, patient understanding of falls risks and participation in care plan implementation did not change and one study showed variable awareness of falls risks. | **Minor concerns:** Six studies used quantitative measures to assess patient engagement or activation (knowledge, skills, and confidence), or participation in care plan development. Two studies reported goals set as part of the intervention and three studies provided rich description using qualitative data about patient and practitioner perspectives. | **Minor concerns:** All studies were in acute settings except one rehab hospital. Five studies were conducted in the US, three in Australia, one in Sweden and one in New Zealand. | **High confidence:** It is highly likely that the review finding is a reasonable representation of the phenomena of interest. | Minor concerns in methods, adequacy and relevance. |
| Where hospital staff are unable to respond quickly to patients’ requests for help, patients may mobilise alone if they feel confident in their ability or are unable to wait due to urgency for the toilet, even if this increases their risk of falling. | [33, 34] | **Minor concerns:** Both studies were qualitative interview studies with clearly described aims and methods. | **Minor concerns:** Both studies reported reasons why patients choose to mobilise alone. | **Minor concerns:** One study included 5 patients and the other included 30. Both studies explored patient perspectives about falls prevention. | **Minor concerns:** Both studies were in acute hospitals. One study was conducted in the UK and one was conducted in the US. | **High confidence:** It is highly likely that the review finding is a reasonable representation of the phenomena of interest | Minor concerns in methods, coherence and relevance. |
| Interventions that encourage cognitively intact patients to participate in falls prevention practices are associated with a reduction in falls. However, it is unclear whether this outcome is because the intervention has led to patient participation in tailored interventions/ goals. | [24, 35, 36, 39-46] | **Minor – moderate concerns**. Seven of the 11 papers were QI studies and differed in the level of detail about methods. Two studies were RCTs and one a non-RCT, one quantitative with clearly described research aims and methods. | **Minor – moderate concerns:**  Five studies evaluated interventions that aimed to involve patients in the assessment and care planning process. Two studies showed a reduction in falls, one a reduction in cognitively intact patients only, one a reduction in units with a higher baseline rate of falls, in one study falls rates varied between hospitals.  Three studies evaluated patient agreements and reported that fall rates declined.    Three studies evaluated intentional rounding. One study reported a reduction in fall rates in one unit, in one study the fall rate did not change, and in one study fall rates increased where purposeful rounding was completed more frequently. | **Minor – moderate concerns**:  Seven studies report falls rates per 1000 bed days, including two RCTs and one non-RCT that evaluated interventions to involve patients in assessment and care planning. A RCT and the non-RCT demonstrated a significant reduction in fall rates. The other RCT found that the intervention reduced falls amongst cognitively intact patients only.  The RCTs and two other studies provide data about goals set and patient engagement (knowledge of falls risks) but not whether patients participated in falls prevention interventions. | **Minor concerns.** All studies were conducted in inpatient settings with adults and included, or focused on, older patients.  Nine studies took place the US and two in Australia. | **High confidence:** It is highly likely that the review finding is a reasonable representation of the phenomena of interest. | Minor concerns in coherence, adequacy and relevance. |
| There is a paucity of literature examining the use of patient participation interventions with cognitively impaired patients. Where available, evidence suggests that, depending on severity of impairment, education, goal setting and follow-up may not lead to participation to support falls prevention. | [30, 36] | **Minor Concerns:**  Study aims and methods clearly described in both studies. One study was a three-group RCT and one was a mixed methods realist evaluation. | **Minor concerns:** Two out of 24 studies reviewed explicitly examined intervention use or impact with patients with cognitive impairment.  One study examined intervention use with mildly impaired patients suggesting reminders prompted participation but fall rates were not measured. One study found that cognitively impaired patients allocated education and physiotherapy follow-up with goal setting incurred a significantly higher rate of injurious falls than participants in the control group. | **Minor concerns:** One study was a 3-group randomised trial that found that cognitively impaired patients allocated education and follow-up incurred a significantly higher rate of injurious falls than participants in the control group. One study included qualitive interviews with patients with mild cognitive impairment. | **Moderate to minor concerns:** studies were conducted in inpatient settings with adults and a rehabilitation hospital. One study took place in Australia and one in New Zealand. | **High confidence**: It is highly likely that the review statement is a reasonable representation of the phenomena of interest. | Minor concerns in methods, coherence, and adequacy. |

1. Carroll, D.L., P.C. Dykes, and A.C. Hurley, *An electronic fall prevention toolkit: effect on documentation quality.* Nursing research, 2012. **61**(4): p. 309-313.

2. Cook, N.S., B.J. Komansky, and M.S. Urton, *Do No Harm: A Multifactorial Approach to Preventing Emergency Department Falls-A Quality Improvement Project.* Journal of emergency nursing, 2020. **46**(5): p. 666-674.

3. Dempsey, J., *Falls prevention revisited: a call for a new approach.* Journal of Clinical Nursing, 2004. **13**(4): p. 479-85.

4. Dowding, D.W., M. Turley, and T. Garrido, *The impact of an electronic health record on nurse sensitive patient outcomes: An interrupted time series analysis.* Journal of the American Medical Informatics Association, 2012. **19**(4): p. 615-620.

5. Healey, F., et al., *Using targeted risk factor reduction to prevent falls in older in-patients: a randomised controlled trial.* Age and Ageing, 2004. **33**(4): p. 390-395.

6. Ireland, S., et al., *Designing a falls prevention strategy that works.* Journal of Nursing Care Quality, 2010. **25**(3): p. 198-207.

7. Koh, S.L.S., et al., *Impact of a fall prevention programme in acute hospital settings in Singapore.* Singapore Medical Journal, 2009. **50**(4): p. 425-432.

8. Maia, F.D.O.M., et al., *Falls prevention strategies for adult inpatients in a university hospital of Sao Paulo, Brazil: A best practice implementation project.* JBI Database of Systematic Reviews and Implementation Reports, 2018. **16**(8): p. 1720-1736.

9. Lytle, K.S., et al., *Clinical Decision Support for Nurses: A Fall Risk and Prevention Example.* Computers, informatics, nursing : CIN, 2015. **33**(12): p. 530-7; quiz E1.

10. McCarty, C.A., et al., *Implementation of the MEDFRAT to Promote Quality Care and Decrease Falls in Community Hospital Emergency Rooms.* Journal of emergency nursing: JEN : official publication of the Emergency Department Nurses Association, 2018. **44**(3): p. 280-284.

11. Ohde, S., et al., *The effectiveness of a multidisciplinary QI activity for accidental fall prevention: staff compliance is critical.* BMC health services research, 2012. **12**: p. 197.

12. Pop, H., et al., *Tailoring a Comprehensive Bundled Intervention for ED Fall Prevention.* Journal of emergency nursing, 2020. **46**(2): p. 225-232.

13. Thatphet, P., et al., *Lessons Learned From Emergency Department Fall Assessment and Prevention Programs.* Cureus, 2021. **13**(7): p. e16526.

14. Townsend, A.B., M. Valle-Ortiz, and T. Sansweet, *A Successful ED Fall Risk Program Using the KINDER 1 Fall RiskAssessment Tool.* Journal of Emergency Nursing, 2016. **42**(6): p. 492-497.

15. Wu, M.W., et al., *Evaluation of Electronic Health Records on the Nursing Process and Patient Outcomes Regarding Fall and Pressure Injuries.* Computers, informatics, nursing : CIN, 2019. **37**(11): p. 573-582.

16. Albornos-Munoz, L., et al., *Falls assessment and interventions among older patients in two medical and one surgical hospital wards in Spain: A best practice implementation project.* JBI Database of Systematic Reviews and Implementation Reports, 2018. **16**(1): p. 247-257.

17. Capan, K. and B. Lynch, *A hospital fall assessment and intervention project.* Journal of Clinical Outcomes Management, 2007. **14**(3): p. 155-160.

18. Milisen, K., et al., *Feasibility of implementing a practice guideline for fall prevention on geriatric wards: a multicentre study.* International Journal of Nursing Studies, 2013. **50**(4): p. 495-507.

19. Titler, M.G., et al., *The effect of a translating research into practice intervention to promote use of evidence-based fall prevention interventions in hospitalized adults: A prospective pre-post implementation study in the U.S.* Applied nursing research : ANR, 2016. **31**: p. 52-59.

20. Businger, A.C., et al., *Lessons learned implementing a complex and innovative patient safety learning laboratory project in a large academic medical center.* Journal of the American Medical Informatics Association, 2020. **27**(2): p. 301-307.

21. Currie, L.M., et al. *Compliance with use of automated fall-injury risk assessment in three clinical information systems*. in *AMIA*. 2006.

22. Duckworth, M., et al., *Assessing the Effectiveness of Engaging Patients and Their Families in the Three-Step Fall Prevention Process Across Modalities of an Evidence-Based Fall Prevention Toolkit: An Implementation Science Study.* Journal of medical Internet research, 2019. **21**(1): p. e10008.

23. Dykes, P.C., et al., *Fall TIPS: strategies to promote adoption and use of a fall prevention toolkit.* Amia .. 2009. **Annual Symposium proceedings / AMIA Symposium. AMIA Symposium. 2009**: p. 153-157.

24. Dykes, P.C., et al., *Pilot Testing Fall TIPS (Tailoring Interventions for Patient Safety): a Patient-Centered Fall Prevention Toolkit.* Joint Commission Journal on Quality and Patient Safety, 2017. **43**(8): p. 403-413.

25. Teh, R.C.A., et al., *Evaluation and refinement of a handheld health information technology tool to support the timely update of bedside visual cues to prevent falls in hospitals.* International journal of evidence-based healthcare, 2018. **16**(2): p. 90-100.

26. Hefner, J.L., et al., *A Falls Wheel in a Large Academic Medical Center: An Intervention to Reduce Patient Falls With Harm.* Journal for healthcare quality : official publication of the National Association for Healthcare Quality, 2015. **37**(6): p. 374-380.

27. Carroll, D.L., P.C. Dykes, and A.C. Hurley, *Patients' perspectives of falling while in an acute care hospital and suggestions for prevention.* Applied Nursing Research, 2010. **23**(4): p. 238-241.

28. Hill, A.-M., et al., *‘My independent streak may get in the way’: how older adults respond to falls prevention education in hospital.* BMJ open, 2016. **6**(7): p. e012363.

29. Kiyoshi-Teo, H., et al., *Older hospital inpatients' fall risk factors, perceptions, and daily activities to prevent falling.* Geriatric nursing (New York, N.Y.), 2019. **40**(3): p. 290-295.

30. Martin, R.A., et al., *Implementing a 'Safe Recovery' fall prevention program: Refining intervention theory using realist methods.* Australasian journal on ageing, 2020. **39**(3): p. e259-e270.

31. Radecki, B., S. Reynolds, and A. Kara, *Inpatient fall prevention from the patient's perspective: A qualitative study.* Applied Nursing Research, 2018. **43**: p. 114-119.

32. Rush, K.L., et al., *Patient falls: acute care nurses' experiences.* Journal of Clinical Nursing (Wiley-Blackwell), 2009. **18**(3): p. 357-365.

33. Turner, N., et al., *The Perceptions and Rehabilitation Experience of Older People After Falling in the Hospital.* Rehabilitation nursing : the official journal of the Association of Rehabilitation Nurses, 2019. **44**(3): p. 141-150.

34. Twibell, R.S., et al., *Perceptions Related to Falls and Fall Prevention Among Hospitalized Adults.* American journal of critical care : an official publication, American Association of Critical-Care Nurses, 2015. **24**(5): p. e78-e85.

35. Christiansen, T.L., et al., *Patient activation related to fall prevention: a multisite study.* The Joint Commission Journal on Quality and Patient Safety, 2020. **46**(3): p. 129-135.

36. Haines, T.P., et al., *Patient education to prevent falls among older hospital inpatients: A randomized controlled trial.* Archives of Internal Medicine, 2011. **171**(6): p. 516-524.

37. Hill, A.-M., et al., *Educators’ perspectives about how older hospital patients can engage in a falls prevention education programme: a qualitative process evaluation.* BMJ open, 2015. **5**(12): p. e009780.

38. Kullberg, A., et al., *Information exchange in oncological inpatient care--patient satisfaction, participation, and safety.* European journal of oncology nursing : the official journal of European Oncology Nursing Society, 2015. **19**(2): p. 142-147.

39. Radecki, B., et al., *Innovating Fall Safety: Engaging Patients as Experts.* Journal of nursing care quality, 2020. **35**(3): p. 220-226.

40. Bargmann, A.L. and S.M. Brundrett, *Implementation of a Multicomponent Fall Prevention Program: Contracting With Patients for Fall Safety.* Military medicine, 2020. **185**(2 Supplementement): p. 28-34.

41. Cann, T. and A. Gardner, *Change for the better: An innovative Model of Care delivering positive patient and workforce outcomes.* Collegian, 2012. **19**(2): p. 107-113.

42. Dykes, P.C., et al., *Fall prevention in acute care hospitals: A randomized trial.* JAMA - Journal of the American Medical Association, 2010. **304**(17): p. 1912-1918.

43. Goldsack, J., et al., *Hourly rounding and patient falls: what factors boost success?* Nursing, 2015. **45**(2): p. 25-30.

44. Johnson, J.E., et al., *Breaking the fall.* Journal of Nursing Administration, 2011. **41**(12): p. 538-545.

45. Vonnes, C. and D. Wolf, *Fall risk and prevention agreement: engaging patients and families with a partnership for patient safety.* BMJ open quality, 2017. **6**(2): p. e000038.

46. Zadvinskis, I.M., et al., *The Impact of Nursing Work and Engagement on Patient Falls.* The Journal of nursing administration, 2019. **49**(11): p. 531-537.
